# A data-driven universal gut microbiome health assessment: a machine learning framework trained on large metagenomic data

**DOI:** 10.3389/fmicb.2026.1925500

**Published:** 2026-09-10

**Authors:** Bablu Kumar, Erika Lorusso, Bruno Fosso, Graziano Pesole

**Affiliations:** 1 Department of Oncology and Hematology-Oncology, Università degli Studi di Milano, Milan, Italy; 2 Department of Biosciences, Biotechnology and Environment, University of Bari A. Moro, Bari, Italy; 3 National Research Council, Institute of Biomembranes, Bioenergetics and Molecular Biotechnologies, Bari, Italy

**Keywords:** dysbiosis, gut microbiome, health-disease classification, machine learning, microbial biomarkers, microbiome-based prediction, shotgun metagenomics

## Abstract

The gut microbiota is essential to maintain host physiology, and its disruption (dysbiosis) is associated with a wide range of diseases. Machine learning (ML) offers a powerful tool to model species-level microbiome profiles, but classifiers that reliably separate healthy from diseased individuals across independent cohorts are still lacking. In this study, we developed a ML classifiers trained on 7,452 publicly available stool metagenomes spanning 32 studies and 12 diseases, designed to distinguish healthy individuals (absence of a clinically diagnosed disease) from non-healthy individuals (presence of a clinically diagnosed disease) based on species-level gut microbiome profiles. We trained 16 supervised models combining four algorithms (RF, SVM-LIN, SVM-RBF, and LR-ElasticNet) combined with all feature sets and three feature-selection algorithms. Performance was assessed by F1 score and ROC–AUC on held-out test data and externally validated on 642 samples from six independent cohorts, including previously unseen diseases. On the test set, all models achieved F1 scores of 78–86% and ROC–AUC values of 89–95%. An SVM-RBF model using permutation-based feature selection performed best (F1 = 86.6%, ROC–AUC = 95.5%; healthy F1 = 86.6%, non-healthy F1 = 88.7%). Importantly, external validation confirmed the generalizability of the full-feature SVM-RBF model (overall F1 = 70.6%; ROC–AUC = 84.7%), including unseen disease types such as *Clostridioides difficile* infection (F1 = 90.3%) and *type 2 diabetes* (F1 = 77.4%). Feature-importance and multivariate analyses revealed both shared and disease-specific microbial signatures, suggesting that the model captures biologically meaningful patterns rather than cohort-specific artifacts. Disease-associated taxa included *Klebsiella pneumoniae*, *Raoultella ornithinolytica, Sutterella wadsworthensis, Gemmiger formicilis*, and *Lactobacillus crispatus*. In contrast, healthy status was consistently associated with commensal species such as *Extibacter hylemonae* and *Ruthenibacterium lactatiformans*. These results show that models trained on pooled metagenomes predict gut health status accurately and transfer to independent cohorts, providing a scalable, non-invasive framework and a set of candidate microbial biomarkers for further clinical evaluation.

## 1 Introduction

The gut microbiota is integral to host physiology, contributing to maintain intestinal homeostasis and host wellbeing (Cho and Blaser, 2012; Fan and Pedersen, 2021; He et al., 2026). The dysbiosis is an alteration of the resident community ecology, and has been associated with several pathological conditions (Fan and Pedersen, 2021; Hou et al., 2022; Van Hul et al., 2024; Joos et al., 2025). Consistent gut microbiota changes across different cohorts support supervised classifiers for identifying disease-specific microbial signatures (Pasolli et al., 2016; Giliberti et al., 2022; Li et al., 2023; Jin et al., 2024; Kumar et al., 2024; Li et al., 2025; Porcari et al., 2025). However, despite a large application of classification models in microbiome field, relying on both machine learning (ML) or Artificial Intelligence (AI) approaches, there is still a gap in training a model recapping a common dysbiosis signature that cuts across multiple conditions.

Shotgun metagenomic allows to sequence total community DNA, enabling species- and strain-level profiling alongside functional characterization, including for uncultured taxa (Quince et al., 2017; Bars-Cortina et al., 2024; Kumar et al., 2024; Visci et al., 2026). This resolution yields the high-dimensional, species-level abundance data required to train models that predict host health status from the whole-community composition rather than a small panel of preselected taxa (Asnicar et al., 2023).

The mechanisms by which microbial communities influence gut homeostasis remain largely uncharacterized, with few experimentally demonstrated causal pathways (Lloyd-Price et al., 2016; de Vos et al., 2022). Indeed, despite consistent microbial patterns recur across different populations, discerning correlation from causation still remain challenging (Wade and Hall, 2019; Basic et al., 2022; Corander et al., 2022; Fonseca et al., 2024).

Several technical factors make metagenomics difficult for conventional statistical methods. Species typically outnumber samples, abundance matrices are zeros inflated, and relative abundances sum to a constant (i.e., data compositionality) (Gloor et al., 2017; Chan and Li, 2024). Further, cohort-specific differences in sampling, DNA extraction, library preparation, and sequencing depth introduce technical variation that mask biological signals when cases and controls are unevenly distributed across studies (Costea et al., 2017; Wirbel et al., 2021). These properties inflate spurious associations (Gloor et al., 2017; Chan and Li, 2024), violate the independence assumptions underlying classical models, and limit the capacity of standard statistical frameworks to capture the nonlinear, higher-order interactions that characterize microbial ecosystems (Sczyrba et al., 2017; Walsh et al., 2023).

In this scenario, the lack of a widely accepted definition of a “healthy” gut microbiome is, in itself, part of the problem, because community composition is shaped by host and environmental factors, that varies both between people and within the same person over time (Hou et al., 2022; Van Hul et al., 2024; Joos et al., 2025; Wang et al., 2026; Zeng et al., 2026). Microbial communities act as complex adaptive ecosystems in which ecological interactions among taxa generate emergent community-level properties (Chang et al., 2023). Functional redundancy is a key: distinct taxa share metabolic niches and co-occur, so very different taxonomic configurations can support similar functions, while similar profiles can yield different functional outcomes (Widder et al., 2016; Tian et al., 2020). Indeed, the metabolic pathways that defining a dysbiotic signature remain poorly characterized (Vieira-Silva et al., 2016; Tian et al., 2020; Zielińska et al., 2025) and the boundary between normal variability in a healthy microbiome and pathological deviation has never been defined (McBurney et al., 2019; Shanahan et al., 2021; Ghosh et al., 2022; Van Hul et al., 2024). Health-associated states are therefore better captured as distributed community structures than as single markers (Zhu et al., 2026), underlining substantial interpersonal variability (Falony et al., 2016). Finally, it remains to be tested whether it is possible to guess host’s health status (i.e., classification) from a single faecal metagenome in the absence of a diagnosis, and whether this inference is generalizable (Gupta et al., 2020; Chang et al., 2024). While functional profiling and longitudinal sampling might address the definition of a eubiotic/dysbiotic signature; the classification challenge can be addressed by leveraging and pooling existing data, such as we propose in the present study.

Machine-learning (ML)-based classifiers are particularly suited to address classification tasks, because they integrate information across many taxa rather than depending on the reproducibility of any individual feature (Goodswen et al., 2021; Casimiro-Soriguer et al., 2022; Asnicar et al., 2023; Bekhet et al., 2025; Karwowska et al., 2025). ML methods can identify such latent community-level signals by modeling nonlinear relationships and higher-order interactions within high-dimensional, sparse, and compositional microbiome data without relying on assumptions of linearity or feature independence (Camacho et al., 2018; Ghannam and Techtmann, 2021; Zhou and Zhao, 2025; Yu et al., 2026).

As larger and more diverse microbiome datasets become available, these approaches can increasingly distinguish robust biological signals from cohort-specific variation (Topçuoğlu et al., 2020; Lee and Lee, 2024; Romano et al., 2025; Pekel et al., 2026). Thus, the objective shifts from identifying individual taxa that characterize health or disease toward predicting host health status from distributed community-level microbial signatures (Gupta et al., 2020; Chang et al., 2024).

Despite the prediction of healthy versus diseased individuals from gut microbiome profiles has been successfully established for different pathologies by using machine-learning models trained on thousands of publicly available microbiome datasets (Thomas et al., 2019; Kraszewski et al., 2021; Chanda and De, 2024; Wu et al., 2024; Zheng et al., 2024; Piccinno et al., 2025; Romano et al., 2025; Yan R. et al., 2025), the reported performance and discriminative taxa substantially differ between these studies. This reflects limited sample sizes, cohort-specific confounding, and methodological heterogeneity (Tierney et al., 2022; Teixeira et al., 2024), affecting models generalizability beyond the study cohort (Li et al., 2025; Barcan et al., 2026).

Pooling cohorts across multiple diseases addresses challenges that cannot be solved even by large cohorts focused on a single disease. (i) It markedly increases the overall sample size, which boosts statistical power and supports more dependable machine (Gupta et al., 2020; Kumar et al., 2024); (ii) it incorporates both biological and technical variability from independent cohorts, enabling the model to detect stable consistent microbial signatures (Li et al., 2025) and (iii) it diminishes the impact of study-specific biases by pushing the model to learn reproducible biological signals (Duvallet et al., 2017); (iv) it provides a broader and more generalizable definition of the healthy population by including individuals from different countries, age groups, and populations (Gupta et al., 2020; Wilmanski et al., 2021; Chang et al., 2024; Huang et al., 2026); (v) it helps reduce diagnosis-related confounding factors, making it easier to distinguish disease-specific effects from changes genuinely associated with dysbiosis (Vujkovic-Cvijin et al., 2020). For instance, Pasolli et al. (2016) demonstrated that cross-study pooling improves phenotype prediction from taxonomic profiles, though on small cohorts. Pooled ML prediction has also been extended to non-intestinal conditions (Giliberti et al., 2022; Pietrucci et al., 2022; Bao et al., 2024; Jin et al., 2024; Lee and Lee, 2024) and recently extend to fungal mycobiome (Defazio et al., 2026). Collectively, these studies show that pooling across cohorts improves the robustness and generalizability of microbiome-based prediction, even if the focus on individual diseases, with only one exception (Defazio et al., 2026).

Taken together, we hypothesize that a supervised ML model trained on species-level relative abundance profiles across multiple disease cohorts’ studies, can classify individuals as healthy (absence of diagnosed disease) or non-healthy (presence of diagnosed disease). Our objective is accurate classification and external validation, not causal inference; feature importance is examined only as a secondary, exploratory analysis to inform future mechanistic work. To test this, we trained a single supervised binary classifier on 7,452 stool shotgun metagenomes from 32 independent studies spanning 12 disease conditions, rather than optimizing for one disease in one cohort. Model selection and evaluation prioritize cross-cohort robustness and external validation over maximal within-dataset performance, directly addressing the generalization failures documented in single-cohort studies. The result is a scalable framework for microbiome-based health prediction with potential relevance to disease screening, independent of any single disease’s specific microbial etiology.

## 2 Materials and methods

### 2.1 Stool-based shotgun metagenomic data collection

We searched PubMed and Google Scholar for the peer-reviewed papers published from inception to 31/08/2024 using the keywords “gut microbiome,” “shotgun sequencing,” “humans,” “stool/faecal,” and “WGS/metagenomes.” We retained studies that primarily focused on stool-based shotgun metagenomic sequencing and include metadata specific to healthy (absence of a diagnosed disease) and non-healthy (presence of a diagnosed disease) individuals, as per the original study. After that, to reduce potential unfairness and to ensure consistency in a large cohort data set, we applied inclusion and exclusion criteria. *Inclusion Criteria* were (i) human stool samples sequenced using shotgun metagenomic sequencing methods on an Illumina platform; (ii) each sample should include information on the health status (i.e., if the individual is healthy or diagnosed with a disease); (iii) at least 10 metagenomes per study. *Exclusion criteria*: (i) samples with lacking information on health status; (ii) data sets generated by non-Illumina platforms, such as Oxford Nanopore, 454 GS FLX Titanium, Ion Torrent PGM, and Ion Torrent Proton. The associated metadata were manually curated from either the Supplementary material or retrieved directly from the Sequence Read Archive (SRA) when not available. For each study, we identified the corresponding *“BioProject ID”* accession and retrieved all associated Run Accession IDs. These Run Accessions were then used to download the raw DNA sequencing reads (FASTQ files) from the NCBI SRA using the SRA Toolkit (fastq-dump).^1^

### 2.2 Preprocessing of shotgun metagenomic sequences

The quality of raw sequencing reads was assessed using FastQC (v0.12.1).^2^ Then, poor quality and adapter regions were trimmed using Fastp (v0.24.0) (Chen et al., 2018) with the following parameters: --detect_adapter_for_pe (to enable paired-end adapter detection), −q 20 (maintain quality score threshold), −u 20 (maximum unqualified base percentage), and -l 45 (minimum read length). After quality filtering, the remaining reads were referred to as ‘*cleaned reads*.’

The cleaned reads were then aligned to the human reference genome (GRCh38)^3^ using Bowtie2 (v2.5.4) with default parameters to remove potential human derived DNA reads contaminants. The --un-con option was used to retain reads that failed to align to the human reference genome, while aligned reads (considered as potentially human DNA reads) were discarded. Only the resulting non-host (unaligned) reads were retained for downstream taxonomic classification.

Taxonomic classification of the retained reads at the species level was performed using Kraken2 (v2.1.3) with default parameters (Wood et al., 2019) against the Unified Human Gastrointestinal Genome (UHGG) v2.0.2 reference database.^4^ This reference database is optimized for prokaryotes and contains 286,997 genomes, representing 4,644 bacteria and archaea species from the human gut (Almeida et al., 2021). To ensure high-quality metagenomes, only samples with >100,000 reads assigned at the species level by kraken2 were retained for downstream analyses.

Next, Bracken (v2.9) was used to correct the relative species abundances (i.e., the proportion of reads assigned to each species) per sample. For Bracken, the Kraken2 report was used as an input with the following parameters: read length (r) = 100 bp, taxonomic level (l) = species (S), and classification threshold (*t*) = 10 reads. Then, using a Python script,^5^ we combined the Bracken result into a single consolidated report to generating a species-by-sample abundance table.

### 2.3 Microbiome diversity analysis

To assess the alpha diversity within groups, the Chao1 estimator and the Shannon index were calculated using the iNEXT R package (v2.0.20) (Hsieh et al., 2016). This package provides standardized estimates of diversity between samples with different sequencing depths through a Hill-number framework based on rarefaction and extrapolation. The Mann–Whitney U test was used to compare alpha-diversity between healthy and non-healthy groups, and between each disease phenotype and the healthy group. The effect size of these differences was assessed using Cliff’s delta (Cliff, 1993). Because diversity estimates obtained from iNEXT did not follow a normal distribution, the non-parametric Mann–Whitney U test was used for all group-level comparisons, consistent with standard practice in large-scale microbiome studies (Wirbel et al., 2019; Bastiaanssen et al., 2023).

To evaluate differences in microbial composition between healthy and non-healthy groups, the Aitchison distance was used, calculated as the Euclidean distance based on centered log-ratio (CLR) transformed species-level abundance data. The analysis was performed using the Python package scikit-bio (v0.5.6). To visualize the compositional similarity and dissimilarity between samples, Principal Coordinate Analysis (PCoA) was performed using the *skbio.stats.ordination.pcoa* function. Additionally, Permutational Multivariate Analysis of Variance (PERMANOVA) was used to test for statistically significant differences in microbial community structure between health status groups and different disease phenotypes. This analysis was performed using the *skbio.stats.distance.permanova* function with 999 permutations.

### 2.4 Batch correction across studies

To correct batch effects arising due to studies cohorts (refers as BioProject), we used the MMUPHin (v1.16.0) package in R, especially designed for zero-inflated microbiome data (Ma et al., 2022). The adjust_batch function was applied to a species-level read count tables (contained number of reads for each species) to adjust for batch effects, specifying “*BioProjectID”* as the batch variable. To measure how much of the variation in microbial composition was explained by BioProject before and after batch correction, Aitchison distance (Euclidean distance) matrices were calculated using the vegdist function from the R package vegan (v2.6.10). Permutational multivariate analysis of variance (PERMANOVA) was then performed with the adonis2 function (999 permutations, by = “terms”) using the formula read_abundance_matrix ~ BioProject. The explained variance (R^2^ values) by *BioProject*, obtained before and after batch correction, were compared to evaluate the reduction of batch effects.

### 2.5 Predictive model development for disease diagnosis

We developed an ML-based predictive model to predict individuals as healthy or non-healthy using species-level gut microbiome profiles from stool metagenomic sequencing. Model development proceeded in four stages: (1) data preprocessing and splitting, (2) baseline training of four supervised algorithms, (3) hyperparameter optimization, (4) feature selection, and (5) evaluation on held out and external data (Section 2.6).

#### 2.5.1 Data preprocessing and splitting

Before model training, the batch-corrected species count matrix was processed as follows:

- To reduce sparsity and the influence of rare species, we applied prevalence-based filtering to the batch-corrected species count matrix. Prevalence was calculated as the percentage of samples in which a given species was detected, where detection was defined as having at least one non-zero read count (Gupta et al., 2020; Cao et al., 2021). To determine the impact of species prevalence filtering on model performance and to choose the optimal filtering threshold, six species-level feature sets were generated using prevalence thresholds from 0 to 25%, evaluated at 5% intervals across the pooled dataset, and each set was benchmarked across all ML models. We retained the 20% threshold (4,022 species), which gave the highest and most stable F1 scores across all models while excluding low-prevalence, sample-specific taxa.
- Following prevalence filtering, the retained species count matrix was CLR-transformed in Python (v3.11.0) using the *skbio.stats.composition.clr* function from the scikit-bio package, to account for the compositional structure of microbiome data (Lee and Lee, 2024; Karwowska et al., 2025; Li et al., 2025). Before transformation, a pseudo-count of 1 was added to all feature counts to handle zero counts in the matrix. This value is the most commonly used choice and ensures that zeros in the input remain zeros after log transformation (Badsha et al., 2020). Each species abundance was then divided by the geometric mean of all species abundances within the same sample, and the natural logarithm of the resulting ratio was taken. Importantly, CLR is a sample-wise transformation that does not use metadata labels and therefore cannot, on its own, introduce information leakage during model training (Austin and Korem, 2025).
- After CLR transformation, the full dataset of 7,452 samples was split in an 80:20 ratio into training (*n* = 5,961; 3,189 non-healthy and 2,772 healthy) and test (*n* = 1,491; 798 non-healthy and 693 healthy) sets. ML models were fitted on the training data, and their predictive performance was evaluated on the held-out test set.

#### 2.5.2 Model algorithms

We evaluated four supervised algorithms: SVM with linear kernel (SVM-Linear), SVM with RBF kernel (SVM-RBF), logistic regression with ElasticNet regularization (LogReg-ElasticNet), and random forest (RF), spanning both linear and non-linear model classes. These algorithms have been commonly applied in microbiome research for modeling disease prediction models and follow the established best practices for microbiome machine learning (Knights et al., 2011; Statnikov et al., 2013; Pasolli et al., 2016; Camacho et al., 2018; Zhou and Gallins, 2019; Topçuoğlu et al., 2020; Goodswen et al., 2021; Greener et al., 2022; Su et al., 2022; Asnicar et al., 2023; Lee and Lee, 2024; Wu et al., 2024).

1. Random Forest (RF): RF is an ensemble-based learning approach that builds multiple decision trees during training, each tree was trained on a randomly sampled subset of the input dataset. The final prediction is obtained by aggregating the output of individual trees through majority voting. Taking advantage of a collection of weak learners, RF improves predictive accuracy and reduces the risk of overfitting compared to a single decision tree. In this study, each tree was trained on species-level features to distinguish microbiome profiles and to identify microbial taxa associated with specific health phenotypes.
2. Support Vector Machine (SVM): The SVM algorithm classifies data by finding the optimal hyperplane that maximizes the margin of separation between target classes in a multidimensional feature space. Previous microbiome-based studies have demonstrated the effectiveness of SVM-based models for analyzing high-dimensional metagenomic datasets, where the number of features exceeds the number of samples (Knights et al., 2011; Lee and Lee, 2024). To determine the most effective Kernel separation configuration for our metagenomic dataset, we evaluated two kernel functions, that is, linear and RBF kernels, with several parameter settings. The RBF kernel captures complex, non-linear relationships by mapping features into a higher-dimensional space, allowing for a linear separator to be defined. On the other hand, the linear kernel identifies the hyperplane that best separates two classes while minimizing classification errors.
3. Logistic Regression with ElasticNet Regularization (LogReg-ElasticNet): Logistic Regression is a widely used supervised learning algorithm for binary classification, modeling the probability of a categorical outcome based on input features. In this study, ElasticNet regularization was applied to take advantage of the strength of both L1 (Lasso) and L2 (Ridge) penalties, thereby enhancing model generalizability. This approach is well-suited for metagenomic data, which often exhibits multicollinearity and sparsity. The L1 component accounts for sparsity by reducing less informative feature coefficients to zero, while the L2 component stabilizes the model by penalizing large coefficients and minimizing the risk of overfitting.

For all four algorithms, the prediction task was identically: each stool metagenome was assigned one binary label, healthy (absence of clinically diagnosed disease) or non-healthy (presence of clinically diagnosed disease). The ML input features were CLR-transformed species-level relative read abundances obtained from UHGG v2.0.2-based taxonomic profiling. Each algorithm was given the same feature matrix and label (healthy/non-healthy), so the models differed only in the learning algorithm, and feature-selection strategy used.

#### 2.5.3 Model hyperparameter tuning

Hyperparameters were tuned on the training set (80% of the data) using a two-step procedure, random search followed by grid search, applied identically to every algorithm (LaPierre et al., 2019; Lo and Marculescu, 2019; van der Sommen et al., 2020).

In the first step, RandomizedSearchCV was used to explore 50 random hyperparameter combinations within predefined search spaces. In the second step, the most promising parameter combinations identified during the random search step were subsequently refined using GridSearchCV to determine the final optimized model configuration. To prevent overfitting, all hyperparameter tuning was performed using 10-fold stratified cross-validation, with the F1-score as the optimization metric. Tested ranges and final optimized values for each model are summarized (Supplementary Table S3).

#### 2.5.4 Evaluation of the model on the test and external validation dataset

The model predictive performances were assessed using three metrics: (1) F1 score (the harmonic mean of precision and recall) (2) the area under the receiver operating characteristic curve (ROC-AUC) (which measures performance across all classification thresholds) as a performance matrix, (3) Class-wise performance was further assessed using the classification report, which provides precision, recall, F1 score, and support for each class (healthy and non-healthy), enabling detailed evaluation of how well the model predict both class. It is important to note that F1 scoring matrices were chosen as our primary metrics in the light of imbalanced datasets, consistent with established practice (Wu et al., 2018; LaPierre et al., 2019; Asnicar et al., 2023).

All models were evaluated on both the held-out test set (20% of the entire dataset) and an independent external validation dataset (642 samples from six independent studies). The external validation dataset was solely used to evaluate model generalizability and was not involved in any phase of model development, including training, testing, and feature selection.

#### 2.5.5 Feature selection algorithm

To identify robust and discriminative microbial biomarkers associated with gut health, three complementary feature selection strategies were applied: (i) Least Absolute Shrinkage and Selection Operator (LASSO), (ii) Permutation Feature Importance (PFI), and (iii) Recursive Feature Elimination with Cross-Validation (RFECV).

1. Least Absolute Shrinkage and Selection Operator (LASSO): Feature selection by LASSO was performed using a logistic regression model with L1 regularization (sklearn.linear_model. LogisticRegression with L1 penalty). The L1 penalty constrains regression coefficients, shrinking less informative features toward zero and thereby enforcing sparsity in the selected feature set. The regularization parameter (α) was optimized over a logarithmic range (‘alpha’: np.logspace(−5, 2, 100)) using both random and grid search within internal cross-validation splits. This approach enables us to select features that contribute most to predictive performance while reducing overfitting (Susin et al., 2020; Ranalli et al., 2023; Garach Vélez et al., 2025).
2. Permutation-based feature importance (PFI) was applied as a model-agnostic methods to quantify the individual contribution of each microbial species to target predictions. This approach assesses the relevance of features by randomly permuting the values of each predictor (species) while maintaining the underlying data structure and then quantifying the resulting change in classification performance. Features that cause a significant decrease in model performance are interpreted as having high predictive importance, while those that have minimal impact on performance are deemed weakly informative or redundant. In this analysis, PFI was implemented using an RF classifier as the base estimator, enabling the estimation of feature importance in a non-parametric and ensemble-based learning context. This configuration enables the capture of non-linear relationships and higher-order interactions among microbial taxa, while also maintaining the model-agnostic interpretability of the resulting importance scores.
3. Recursive Feature Elimination with Cross-Validation (RFECV): RFECV was used to identify an optimal subset of features for predicting the health status via iterative feature elimination. This approach is based on repeatedly fitting a base estimator and systematically removing the least informative feature(s) at each iteration (Sanz et al., 2018). Here, a trained linear kernel of SVM (SVM-Linear) acts as the base estimator. The linear kernel was chosen to keep the model complexity low, which is advantageous when the sample size is limited but the feature space is high-dimensional. Feature importance was derived from the magnitude of the model coefficients (weight vectors), and at each iteration the feature with the lowest absolute contribution to the decision function was eliminated. This recursive process was continued until an optimal feature subset was identified. Optimality was operationally defined as the feature set that maximized the F1 score across 100 bootstrap iterations of internal train–test resampling, thereby ensuring robustness, stability, and reproducibility of the selected feature panel.

### 2.6 Multivariate analysis of factors contributing to machine learning models utilizing MaAsLin2

Multivariable association analyses were performed using the MaAsLin2 (v 1.16.0) package in R^6^ to assess biological relevance of ML derived features (Mallick et al., 2021). The analysis included the top 50 microbial species ranked by their importance scores in the ML models. Default MaAsLin2 parameters were used, except that healthy individuals were defined as the reference group, following recent recommendations (Nearing et al., 2022; Yang and Chen, 2023). MaAsLin2 applies a linear model to the transformed abundance of each feature across sample groups, estimates significance using a Wald test, and adjusts *p*-values for multiple testing using the Benjamini–Hochberg false discovery rate (FDR) method. The resulting output “significant_results.tsv” file was used to selectively retain statistically significant species for visualization, thus improving heatmap clarity and interpretability. The filtered data were imported into R, and heat maps were generated using the pheatmap package (v1.0.12).

### 2.7 SHAPley values inference

To interpret the SVM-RBF model, SHAP (SHapley Additive exPlanations) values were computed by using the Python library SHAP (v0.51.0) (Lundberg and Lee, 2017). To address the complexity of non-linear kernel and cope with computational requests the algorithm *permutation* was employed in the KernelExplainer class and shap values inference was parallelized among samples by using 100 chunks on 100 CPU each accessing to 100 Gb memory. Globally feature importance was measured as the mean absolute SHAP value across all samples, while the average signed SHAP value was used to assess the direction of the contribution toward each class. Features with positive mean SHAP values were interpreted as promoting the Healthy class, whereas negative values indicated an association with the Non-Healthy class. Only features with a mean SHAP values greater than zero were retained.

## 3 Result

### 3.1 Overview of the data collection and reprocessing using bioinformatics pipelines

**Figure 1:**
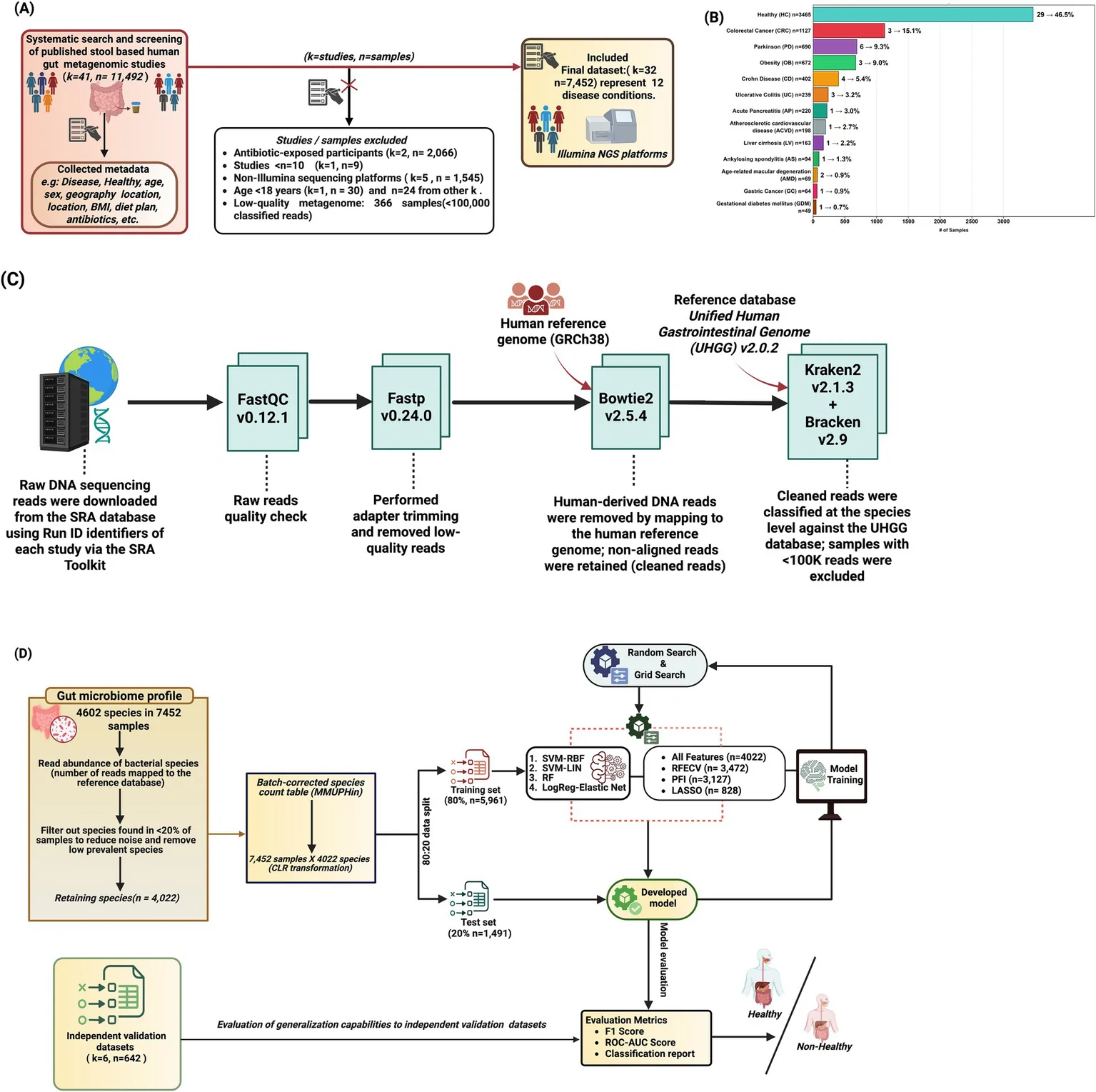
A schematic representation of the computational workflow that includes the retrieval of shotgun metagenomic data derived from stool, the preprocessing procedures, and the development of machine learning models. **(A)** Shotgun metagenomic datasets derived from stool for multiple disease phenotypes were systematically retrieved from PubMed and Google Scholar. The flow chart shows, at each step, the number of studies considered, excluded, and included in the analysis. **(B)** The horizontal bar plot provides a summary of 7,452 human gut metagenomic samples obtained from 32 independent studies. Each bar is color-coded according to one of 12 disease types, including a healthy control group, and is ranked by sample size. For each category, the relative proportion of samples and the number of contributing studies are indicated in parentheses. **(C)** Raw sequencing reads were retrieved and subsequently re-analyzed using a standardized bioinformatics workflow specifically designed in this study, to generate species-resolved microbial read abundance tables. **(D)** An ML framework to classify the health status of the gut (healthy vs. non-healthy) using the gut microbiome profiles at the species level. The pipeline applies a prevalence-based method to filter out low-prevalence species (<20%), batch-effect correction, and centered log-ratio (CLR) transformation. The data were split 80:20 into training (*n* = 5,961) and test (*n* = 1,491) sets. Three feature-selection strategies were then used, and models were trained with systematic hyperparameter optimization via randomized and grid search. The optimized model was first evaluated on the held-out test set. Model generalization capability was further assessed on six independent validation studies (*n* = 642). Performance was measured using F1 score, ROC-AUC, and class-wise classification reports for distinguishing healthy from non-healthy individuals.

To train a machine learning model able to predict host health status based on gut microbiome profiles, we collected a total of 11,492 publicly available stool-derived shotgun metagenomes from 41 studies. Following application of predefined inclusion criteria (Methods and Figures 1A,C), 7,452 metagenomes from 32 studies, encompassing 12 distinct disease conditions, were retained for downstream analysis. Criteria for metagenome exclusion are detailed (Supplementary Section 1.2; Supplementary Table S1). For each included study, associated metadata such as sample size, disease classification, and geographical origin were manually curated and are summarized in Supplementary Table S2.

Based on the health status reported in the original studies, metagenomes were stratified into two classes: non-healthy (*n* = 3,987; 53.6%) and healthy (*n* = 3,465; 46.4%) (defined as the presence and absence of clinically diagnosed specific disease). Within the non-healthy group, major disease categories included colorectal cancer (CRC; *n* = 1,132; 15.1%), Parkinson’s disease (PD; *n* = 690; 9.3%), obesity (OB; *n* = 672; 9.0%), Crohn’s disease (CD; *n* = 402; 5.4%), ulcerative colitis (UC; *n* = 293; 3.2%), and type 2 diabetes (T2D; *n* = 164; 1.8%), with additional disease categories summarized in Figure 1B. We acknowledge that the individuals classified as healthy were defined solely by the absence of a diagnosed disease according to the original studies. This definition may not account for undiagnosed comorbidities, microbiome-modulating medications (e.g., antibiotics, proton pump inhibitors), or demographic and dietary heterogeneity across cohorts, which may introduce label noise into the healthy class.

We downloaded raw sequencing reads (FASTQ format) from the SRA database by using the “run accession IDs” for all selected studies. These raw reads were reprocessed and taxonomically profiled at the species level using a unified bioinformatics pipeline (Figure 1C), ensuring consistency across all datasets and minimizing technical variability introduced by differences in original processing workflows. Across all metagenomic datasets, we identified a total of 4,602 bacterial species, with an average of 2,620 species per sample, highlighting the diverse nature of gut microbial communities (Supplementary Section 1.3; Supplementary Figures S1A,B; Supplementary Table S3). We focused exclusively on bacterial species, as bacteria constitute the major component of the gut microbiome (Staudacher and Loughman, 2021; de Vos et al., 2022) and species-level resolution provides the most accurate and complete representation for discriminating disease-associated microbial signatures compared to broader taxonomic ranks (Giliberti et al., 2022; Lee and Lee, 2024; Van Hul et al., 2024).

To characterize baseline differences between healthy and non-healthy individuals, we assessed alpha diversity (Shannon and Chao1 indices) and beta diversity (Aitchison distance) across 7,452 samples (3,465 healthy, 3,987 non-healthy) (Supplementary Section 1.4). Both Shannon and Chao1 were significantly higher in healthy individuals (*p* = 2.07 × 10^−14^ and *p* = 4.16 × 10^−5^, respectively, Supplementary Figures S2A,C), but effect sizes were small (Cliff’s Δ = 0.103 and 0.055), and the two indices did not always move in the same direction across the 12 disease phenotypes—for example, Shannon diversity decreased in Crohn’s disease but increased in colorectal cancer. In contrast, Chao1 richness showed the opposite pattern in several conditions (Supplementary Figures S2B,D; Supplementary Table S4), consistent with disease-specific rather than uniform shifts in community diversity. Similarly in beta diversity, PERMANOVA test on Aitchison distance showed a statistically significant but modest separation between healthy and non-healthy groups (R^2^ = 0.56%, *p* = 0.001, Supplementary Figure S3A), whereas phenotype-level classification across all 12 diseases explained substantially more variance (R^2^ = 4.04%, ~7-fold higher, Supplementary Figure S3B), with Crohn’s disease, liver disease, and colorectal cancer showing the largest separation (Supplementary Table S5)—indicating that disease-specific compositional signal exists but is diluted when diverse conditions are pooled into a single non-healthy category. In sum, both alpha and beta diversity analysis results indicate that conventional diversity metrics capture only modest, disease-heterogeneous signal (Williams et al., 2024).

Furthermore, because the pooled dataset comprised samples from multiple independent studies, microbial profiles varied with study cohort, laboratory protocol, sequencing platform, and geographic origin. Such heterogeneity can adversely affect model performance (Ma et al., 2022; Kumar et al., 2024). We therefore applied MMUPHin to correct batch effects, using BioProject ID as the batch variable. We assessed the effect of this correction using PERMANOVA based on Bray–Curtis dissimilarities before and after correction, evaluating both study of origin and health status as explanatory variables (Supplementary Section 1.5; Supplementary Figures S4A,B). Before correction, study of origin explained 13.0% of microbial community variation in the training set and 10.4% in the external validation set (PERMANOVA R^2^ = 0.130 and 0.104, respectively; *p* = 0.001 for both), whereas health status explained only 0.10% (R^2^ = 0.00101, *p* = 0.001) and 0.51% (R^2^ = 0.00514, *p* = 0.001) of the variation, respectively. After correction, study-driven variance fell by approximately half, to 6.8 and 5.8% (R^2^ = 0.068 and 0.058), while the variance explained by health status remained stable or slightly increased (training 0.09%, R^2^ = 0.00092; validation 0.61%, R^2^ = 0.00608; *p* = 0.001), confirming preservation of disease-associated microbial signatures. Throughout, *“*training *datasets”* denotes the pooled cohorts split 80:20 into training and test subsets, and “external validation set” denotes the independent cohorts, held out entirely from model development, on which final performance was assessed.

### 3.2 Development of binary classification machine learning models leveraging stool-derived microbiome data

Given the growing potential of the gut microbiome for disease prediction and the development of machine learning-based diagnostic frameworks (Lee and Lee, 2024; Lee et al., 2024; Liu et al., 2024; Li et al., 2025; Tegegne and Savidge, 2025), we trained and evaluated four machine learning models — SVM-RBF, SVM-Linear, Logistic Regression with ElasticNet regularization (LogReg-ElasticNet), and Random Forest (RF) to develop a predictive model to predict healthy (absence of diagnosed disease) and non-healthy (presence of diagnosed disease) using microbial species profiles obtained from stool-based shotgun metagenomic datasets (Figure 1D).

Before model training, we applied prevalence-based species filtering (referred to as the presence/absence of taxa) to reduce noise from low-prevalence and sample-specific taxa in overall metagenomic datasets. Species were retained based on the presence/absence prevalence thresholds ranging from 0 to 25%, evaluated systematically to identify the optimal filtering threshold for downstream classification (Supplementary Figure S6A). Following centered log-ratio (CLR) transformation on filtered species and their read counts, the modeling datasets was then randomly split to form a training (80%) and hold-out test (20%) sets. All four models were trained on training datasets (healthy *n* = 2,772; non-healthy *n* = 3,189), and their predictive performance was evaluated on test datasets (healthy *n* = 693; non-healthy *n* = 798) (Figure 1D).

Benchmarking model performance across prevalence thresholds revealed that retaining species present in ≥20% of samples (*n* = 4,022) produced the most stable and reproducible predictive performance across all classifiers, with F1 scores consistently exceeding 79.4% (Supplementary Section 1.7; Supplementary Figure S6B). This threshold was therefore selected for all downstream analyses, and the resulting feature set is hereafter referred to as the “*all-feature set*.”

**Figure 2:**
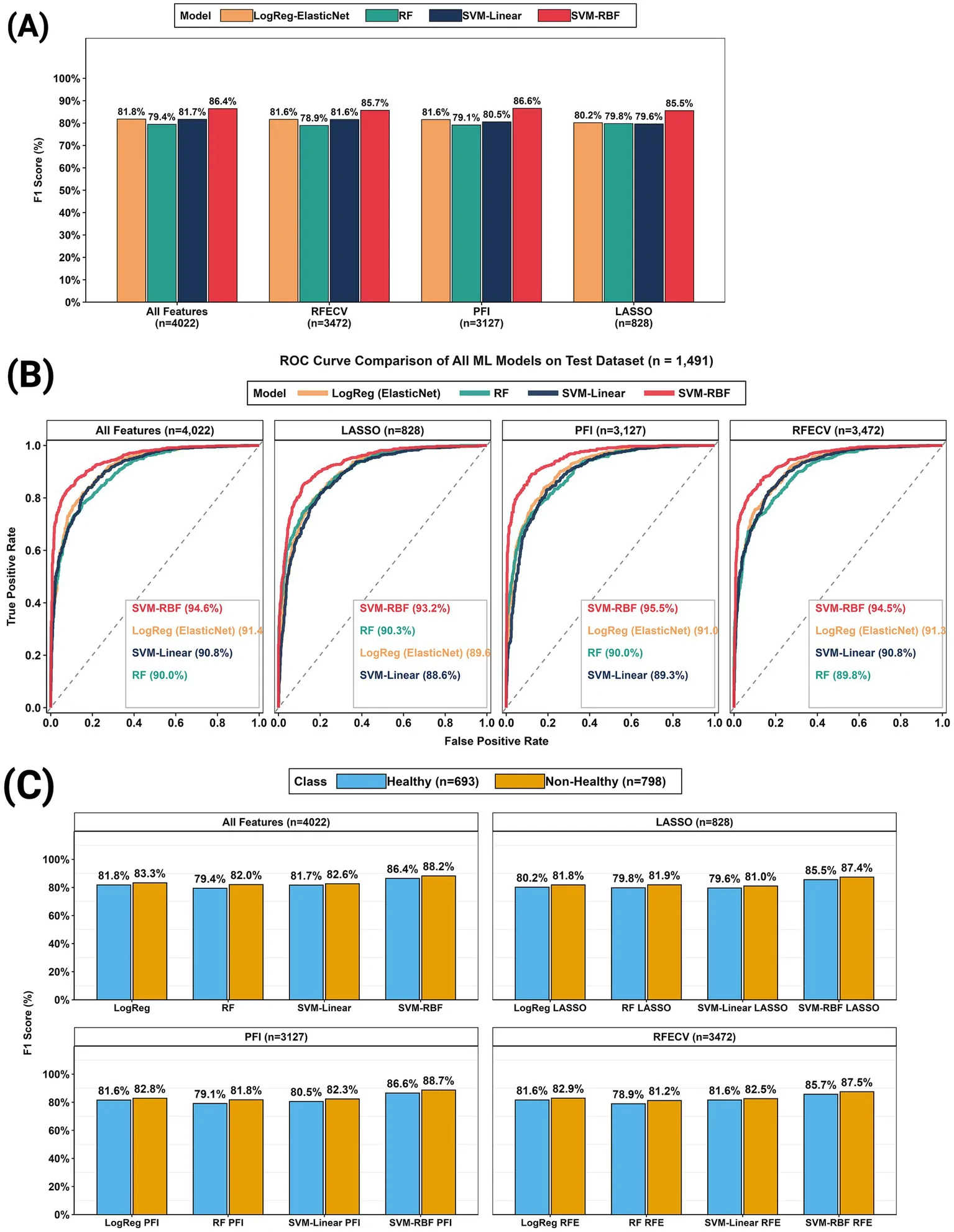
Performance comparison of sixteen ML models that include four classifiers (SVM-RBF, SVM-Linear, RF, and LogReg-ElasticNet)—trained on full feature set (4,022 species) and three feature-selection strategies: LASSO (828 species), PFI (3,127 species), and RFECV (3,472 species). Model performance was assessed on a test dataset (total *n* = 1,491; healthy: *n* = 693; non-healthy: *n* = 798) using F1 score and ROC-AUC metrics as performance metrics. **(A)** Grouped bar plots summarize overall F1 scores across models’ configurations (denoted above the bar); SVM-RBF-PFI yielded the highest F1 scores. **(B)** ROC curves illustrate discrimination performance by feature subset, with ROC-AUC values (%) reported for each classifier. The grey dashed diagonal indicates random performance (AUC = 0.50); curves closer to the upper left corner indicate better discrimination. **(C)** Grouped bar plots report class-specific F1 scores for healthy and non-healthy individuals, stratified by feature-selection method. Bars are colored by class (healthy: sky blue; non-healthy: golden yellow), with F1 scores annotated. In **(A,B)**, classifiers are color-coded: SVM-RBF (crimson red), SVM-Linear (deep navy blue), RF (teal green), and LogReg-ElasticNet (warm orange).

Next, using the trained model on the *all-feature set*, we observed that the SVM-RBF model outperformed all other evaluated models. In terms of overall performance, on the test dataset, it achieved an F1 score of 86.4% (Figure 2A) and a ROC–AUC of 94.6% (Figure 2B). Class-specific performance was similarly best, with F1 scores of 88.2 and 86.4% for the non-healthy and healthy classes, respectively (Figure 2C). Additionally, other models demonstrate competitive performance but consistently had lower F1 score than the SVM-RBF model. The LogReg-ElasticNet model achieved an F1 score of 81.8% and ROC–AUC of 91.4% (Figures 2A,B). Similarly, the SVM-Linear model with an F1 score of 81.7% and an ROC–AUC of 90.8%, while the RF model achieved an F1 score of 79.4% and an ROC–AUC of 90.0% (Figures 2A,B). Class-wise performances across healthy and non-healthy groups are summarized in Figure 2C, demonstrating comparatively lower predictive performance of all evaluated models compared to the SVM-RBF classifier. A comprehensive summary of all the other classification metrics, such as balanced accuracy, overall accuracy, precision, and recall, for all models, is provided in Supplementary Table S7.

Overall, the SVM-RBF model demonstrated the best and most consistent predictive performance across both healthy and non-healthy classes, outperforming all other evaluated classifiers in discriminating gut health status from species-level microbial profiles. We speculated that this model not only accurately captures disease-associated microbial changes but also effectively identifies stable and consistent microbial signatures present in healthy individuals. Consequently, all subsequent analyses were conducted using the SVM-RBF model to facilitate further comparisons.

To assess whether predictive performance could be enhanced by reducing the dimensionality of the original subset of features, the SVM–RBF model was retrained on the feature subset of the top 50 microbial species (Supplementary Table S8). Focusing on this reduced set was intended to prioritize the most informative and putatively influential predictors of health status. These species were selected directly from the original SVM–RBF model, ranked by their feature importance scores. However, we found that the retrained model exhibited reduced performance, the overall F1 score declined to 77.6%, and the ROC–AUC decreased to 88.2% on the test set (Supplementary Figure S8). We speculated that the decrease in performance is due to the exclusion of additional informative species. This indicates that feature ranking based solely on marginal importance scores is insufficient to capture the full complexity of health- and disease-associated microbial signatures and inter-species co-dependencies within the gut microbiome. The collective predictive signal is distributed across a broader feature space than any single ranking metric can identify (Layeghifard et al., 2017; Coyte and Rakoff-Nahoum, 2019; D’Elia et al., 2023). To overcome this limitation and improve predictive accuracy, we next sought to apply machine learning–based feature selection strategies to more effectively identify the most informative gut microbial species, followed by retraining all models on each optimized feature subset. This approach also offers an effective way to select microbial biomarkers and address the intrinsic sparsity of microbiome data, as previously recommended (Asnicar et al., 2023).

### 3.3 SVM-RBF with PFI based feature selection methods improved predictions of human gut health

To enhance overall predictive performance and reduce the dimensionality of the metagenomic feature space, we applied three feature selection techniques: RFECV, PFI, and LASSO (see Method 2.5.5). Each method systematically reduces the dimensionality of the metagenomic feature space by retaining the most informative variables while discarding less discriminative ones, thereby improving model efficiency, interpretability, and robustness (Garach Vélez et al., 2025). Utilizing all three feature-selection approaches, RFECV retained 3,472 species, PFI retained 3,127 species, and LASSO selected 828 species. All four base classifiers were then retrained on each of the three feature-selected subsets, resulting in twelve further model configurations: RF-PFI, RF-RFECV, RF-LASSO, SVM-RBF-PFI, SVM-RBF-RFECV, SVM-RBF-LASSO, SVM-LIN-PFI, SVM-LIN-RFECV, SVM-LIN-LASSO, LogReg-ElasticNet-PFI, LogReg-ElasticNet-RFECV, and LogReg-ElasticNet-LASSO. During retraining, we applied the identical procedure as used for the original “all-feature” configuration, thereby maintaining methodological consistency and enabling fair and robust comparison across all model configurations.

Among all retrained models, the SVM-RBF model trained on PFI-based features (SVM-RBF-PFI, *n* = 3,127 species) demonstrated the best overall performance on the hold-out test dataset, achieving the highest F1 score of 86.6% (Figure 2A) and a ROC–AUC of 95.5% (Figure 2B). This performance was marginally superior to the SVM-RBF model trained on the full feature set (F1 = 86.4%), indicating that PFI-based feature selection preserved and slightly improved the discriminative signal of the complete microbial profile while reducing feature dimensionality. Class-wise evaluation further demonstrates that consistent improvement in predictive performance across both classes, with an F1 score of 86.6% for the healthy group and 88.7% for the non-healthy group (Figure 2C). These observations suggest that PFI effectively identifies a compact yet highly informative subset of microbial features that captures the most discriminative biological signals associated with host health status.

For other feature selection methods, the SVM-RBF-RFECV model achieved an F1 score of 85.7% and a ROC-AUC of 94.5%, slightly lower than the PFI-based model. The SVM-RBF-LASSO model achieved an F1 score of 85.5% and a ROC-AUC score of 93.2% (Figures 2A,B). While LASSO achieved comparable performance with substantially fewer features, the broader PFI feature set may better capture complex microbial interactions and community-level dynamics relevant to health status, consistent with the known ecological redundancy of the gut microbiome (Coyte and Rakoff-Nahoum, 2019). For LogReg-ElasticNet, both PFI and RFECV configurations showed identical performance, each achieving an F1 score of 81.6% and a ROC-AUC score of 91% (Figures 2A,B). The LogReg-ElasticNet-LASSO model underperformed slightly, with an F1 score of 80.2% and an ROC-AUC score of 89% (Figures 2A,B). Similarly, SVM-Linear models followed the same trend. The SVM-LIN-RFECV model attained an F1 score of 81.6% and a ROC-AUC score of 88%, followed by SVM-LIN-PFI with an F1 score of 80.5% and a ROC-AUC score of 89%, and SVM-LIN-LASSO with an F1 score of 79.6% and a ROC-AUC score of 88.6% (Figures 2A,B). RF models performed comparatively lower compared to all other models. The RF-LASSO model achieved an F1 score of 79.8% and a ROC-AUC score of 90.3%, followed by RF-PFI with an F1 score of 79.1% and a ROC-AUC score of 89.3%, and RF-RFECV with an F1 score of 78.9% and a ROC-AUC score of 89.8%. Class-wise performance of all models is provided (Figure 2C).

In sum, across all sixteen models, including those trained on the full feature set, the SVM-RBF model with PFI-based feature selection (i.e., SVM-RBF-PFI) exhibited the best predictive performance on the test dataset for classifying gut health status. PFI-based feature selection outperformed both LASSO and RFECV; it is likely that PFI effectively identified most discriminative microbial taxa, reducing the dimensionality of the datasets while improving model accuracy. It also captures the complex relationships between gut microbiome composition and host health status, without excessive dimensionality reduction (Papoutsoglou et al., 2023; Garach Vélez et al., 2025). Consequently, PFI-based selection outperformed alternative feature selection strategies in capturing microbial signals relevant to health status prediction.

### 3.4 Identification of key gut microbiome species and the use of MaAsLin2 to enhance biological interpretability in disease phenotypes

**Figure 3:**
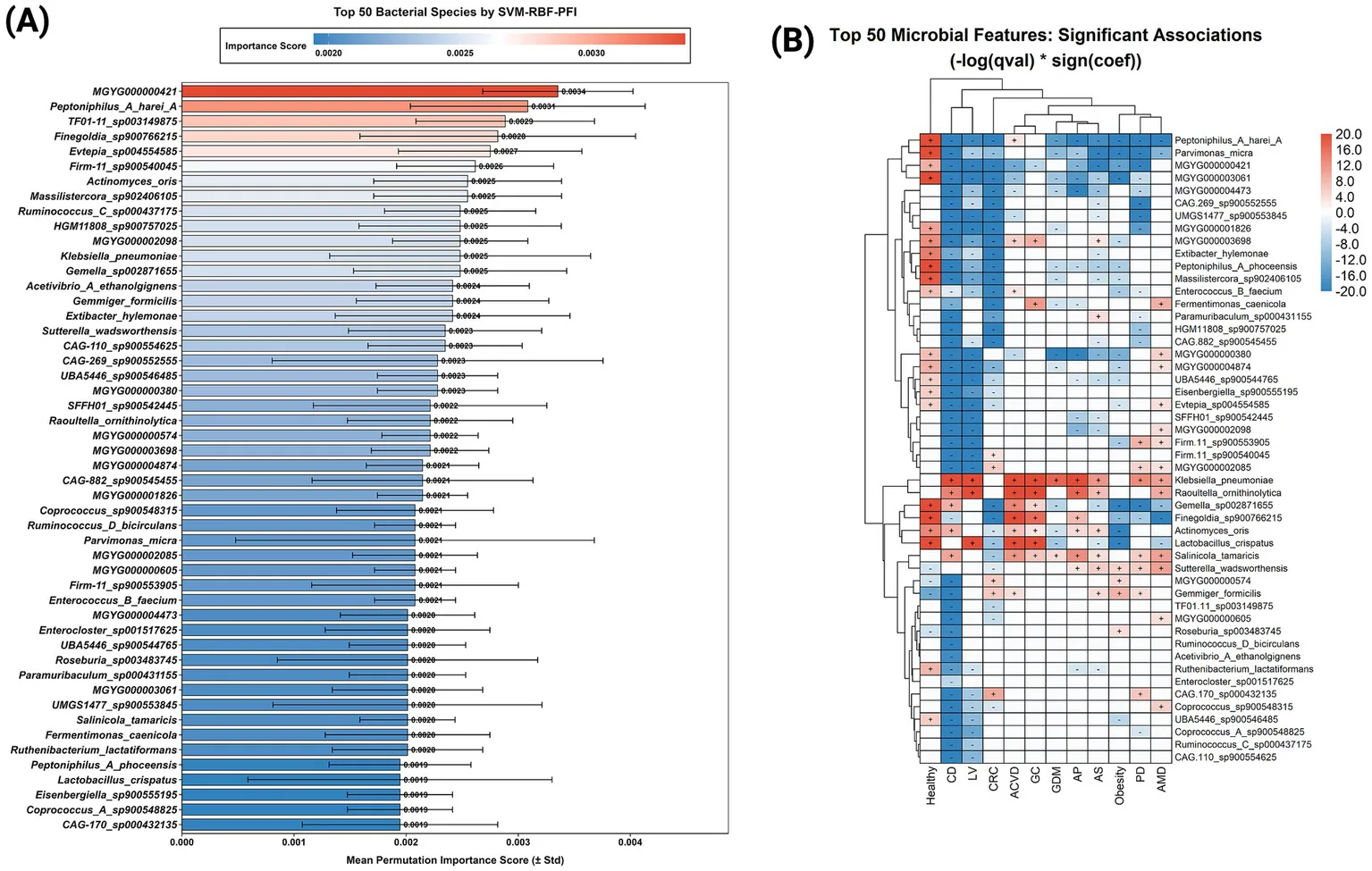
Identification and biological validation of top predictive gut microbial species associated with host health status. **(A)** The horizontal bar plot shows the top 50 gut microbial species ranked by permutation importance derived from the SVM-RBF-PFI model. Species are ordered from highest (top) to lowest (bottom) importance along the y-axis. Bar colors follow a blue gradient (light blue indicates lower importance, medium blue for moderate importance, and dark blue for highest importance) based on mean importance values. Error bars show the standard deviation of importance scores across iterations, reflecting the stability and reliability of each species contribution to model predictions. **(B)** Heatmap illustrating multivariable association analysis using MaAsLin2 reveals the top 50 gut microbial species significantly associated with healthy and distinct disease phenotypes. Rows correspond to phenotypes (including healthy and disease individual conditions), and columns correspond to microbial species. Both dimensions were hierarchically clustered using Euclidean distance to identify coherent association patterns across taxa and phenotypes. Associations are displayed using a continuous color scale representing transformed significance values (−log₁₀(*q*-value) multiplied by the sign of the regression coefficient), ranging from −20 to 20. Positive associations are shown in red (with darker shades indicating stronger effects), negative associations are shown in blue, and non-significant associations are shown in white. Associations achieving statistical significance after Benjamini–Hochberg false discovery rate correction (FDR < 0.05) are annotated with “+” (positive association) or “−” (negative association). ACVD, acute cardiovascular disease; AMD, age-related macular degeneration; AP, acute pancreatitis; AS, ankylosing spondylitis; CD, Crohn’s disease; CRC, colorectal cancer; GC, gastric cancer; LD, liver disease; PD, Parkinson’s disease; UC, ulcerative colitis. Full statistical results are provided in Supplementary Table S9.

To identify the microbial predictors driving model performance and evaluate their biological relevance, we used the best-performing model, SVM–RBF–PFI, to rank gut microbial species in the overall test dataset according to their contribution to health-status prediction. We focused subsequent analyses on the top 50 species that were consistently ranked across the overall test dataset (Figure 3A), as a result yielding a prioritized set of predictive taxa.

Because PFI ranking reflects predictive importance but does not directly estimate the direction or strength of association with health status, we conducted complementary multivariate association testing using MaAsLin2 (v.1.26.0). MaAsLin2 applies generalized linear models to identify statistically significant associations between microbial taxa and host phenotypes while controlling for covariates and false discovery, an approach previously applied in large-scale metagenomic studies to validate machine learning-derived microbial signatures (Su et al., 2022; Bao et al., 2024; Lee and Lee, 2024; Lee et al., 2026). This integrated strategy, combining model-based importance ranking with MaAsLin2-derived association testing, ensures that the identified taxa are not only key contributors to predictive performance but also exhibit statistically robust and biologically relevant associations with health and disease phenotypes.

By using MaAsLin2-based multivariate analysis, we found significant associations between gut microbial species and health status phenotypes (FDR < 0.05, Benjamini–Hochberg correction). In total, we identified 94 positive associations across 37 species (Figure 3B; Supplementary Table S9). Among these, *Klebsiella pneumoniae* demonstrated the broadest spectrum of associations, showing positive correlations with nine disease phenotypes (PD, CD, AP, AMD, GDM, GC, ACVD, LV, AS). Several other taxa demonstrated multi-phenotype relevance such as *Salinicola tamaricis* (eight phenotypes- ACVD, AMD, AP, AS, CD, GC, GDM, PD), *Raoultella ornithinolytica* (seven phenotypes- ACVD, AMD, AP, AS, CD, GC, LD), *Actinomyces oris* (six phenotypes- Healthy, ACVD, AP, AS, CD, GC), *Sutterella wadsworthensis* (five phenotypes- AMD, AP, AS, Obesity, PD), and *Gemmiger formicilis* (five phenotypes- ACVD, AS, CRC, Obesity, PD).

Interestingly, we also found that a subset of microbial species was associated with both healthy and disease phenotypes (LV, ACVD, GC), including *Gemella sp002871655, Finegoldia sp900766215, Lactobacillus crispatus, MGYG000003698, and Peptoniphilus_A harei_A.*

In addition, several taxa were significantly associated with healthy individuals, including *MGYG000003061, Peptoniphilus_A phoceensis, Massilistercora sp902406105, Extibacter hylemonae*, and *Ruthenibacterium lactatiformans*, suggesting further validation required for the candidate biomarkers of healthy gut status. Another species, *Parvimonas micra,* was identified among taxa associated with healthy individuals in our dataset.

Overall, our findings emphasize the biological relevance of the SVM-RBF-PFI model and demonstrate its robustness in identifying gut microbial signatures associated with both physiological and pathological states. These signatures may serve as reliable predictive biomarkers, highlighting the need for validation before any identified taxa are proposed as clinical biomarkers.

### 3.5 Evaluation of SVM-RBF model generalization capability on external validation cohorts

**Figure 4:**
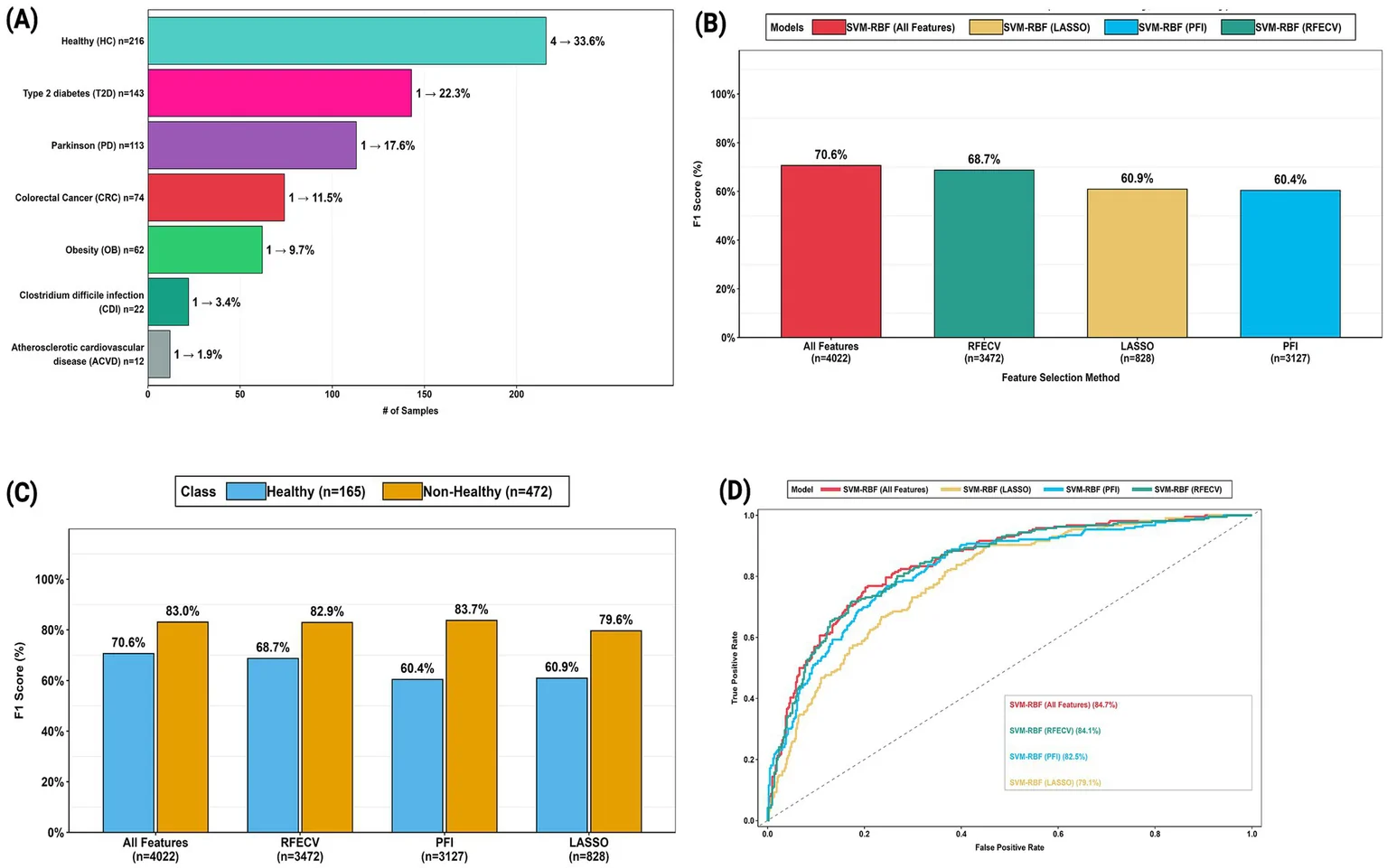
Overview of the validation dataset (*n* = 642 from six studies) and evaluation of the generalization performance of SVM-RBF models trained on all features, RFECV, LASSO, and PFI using F1 score and ROC-AUC score. **(A)** A horizontal bar graph displays the total number of samples in the validation dataset, with bar colors representing disease type and labels above each bar indicating the contributing study and sample percentage. **(B)** Overall F1 score comparison across four SVM-RBF model configurations, each trained using a distinct feature set: complete feature space (4,022 species), LASSO-selected features (828 species), PFI-selected features (3,127 species), and RFECV-selected features (3,472 species). Bars are color-coded by feature selection method, with F1 percentages annotated above each bar. **(C)** A grouped bar plot shows class-specific F1 scores, with bars grouped by feature-selection method and colored by class (healthy blue, *n* = 165; non-healthy orange, *n* = 472). **(D)** ROC–AUC curves compare the classification performance of all SVM-RBF models trained on all features (red), RFECV (teal), PFI (cyan), and LASSO (yellow). The diagonal dashed line represents random performance (AUC = 0.5), and ROC–AUC values are shown at the bottom right, color-coded to match each curve.

To this end, we found that our SVM–RBF model, trained on all features and incorporating feature selection, generally outperformed all other models within the test dataset. Next, to evaluate how well they generalize to previously unseen data, we assessed their performance on independent metagenomic datasets, hereafter referred to as the validation dataset. In the validation datasets, we obtained a total of 642 stool-derived metagenomes from six published studies (associated metadata are provided in Supplementary Table S10). These datasets included samples from six disease conditions: type 2 diabetes (T2D, *n* = 127), Parkinson’s disease (PD, *n* = 96), Colorectal Cancer (CRC, *n* = 70), Obesity (OB, *n* = 61), Clostridioides Difficile Infection (CDI, *n* = 22), and Atherosclerotic Cardiovascular Disease (ACVD, *n* = 12) (Figure 4A). Notably, the CDI and T2D disease datasets represent a unique case, as neither this disease type nor the corresponding studies were represented in the training dataset. All validation metagenomes were reprocessed through the same unified bioinformatic pipeline applied to the training data to minimize methodological biases (*Methods Section 2.2*). Importantly, all validation data were excluded from model training, internal cross-validation, and feature selection. This design ensured cohort-level independence and prevented data leakage, thereby enabling an unbiased evaluation of the model’s generalization performance.

On the external validation datasets, the SVM–RBF model trained on all sets of features demonstrated the highest overall performance, achieving an F1 score of 70.64% and an ROC–AUC of 84.7% (Figures 4B,D; Supplementary Table 11). Class-wise evaluation revealed significant performance for both healthy individuals (F1 = 70.6%) and non-healthy individuals (F1 = 83%) (Figure 4C). In comparison, the SVM–RBF–RFECV model showed slightly lower performance, with an overall F1 score of 68.72% and an ROC–AUC of 84.1%, and class-specific for healthy individuals (F1 = 68.7%) and for non-healthy individuals (F1 = 82.9%) (Figure 4C). The SVM–RBF–PFI model demonstrated lower performance, with an overall F1 score of 60.43% and an ROC–AUC of 82.5% (Figures 4A,D). While this model showed marginally improved performance of non-healthy samples, achieving an F1 score of 83.7%. However, its performance on healthy samples declined substantially (F1 = 60.4%). Similarly, the SVM–RBF–LASSO model achieved an overall F1 score of 60.91% and an ROC–AUC of 79.1%. Class-wise performance for other models is provided in Figure 4C. Such performance from an independent validation cohort further confirmed the robustness and generalizability of our SVM-RBF with all features model across different disease datasets.

**Figure 5:**
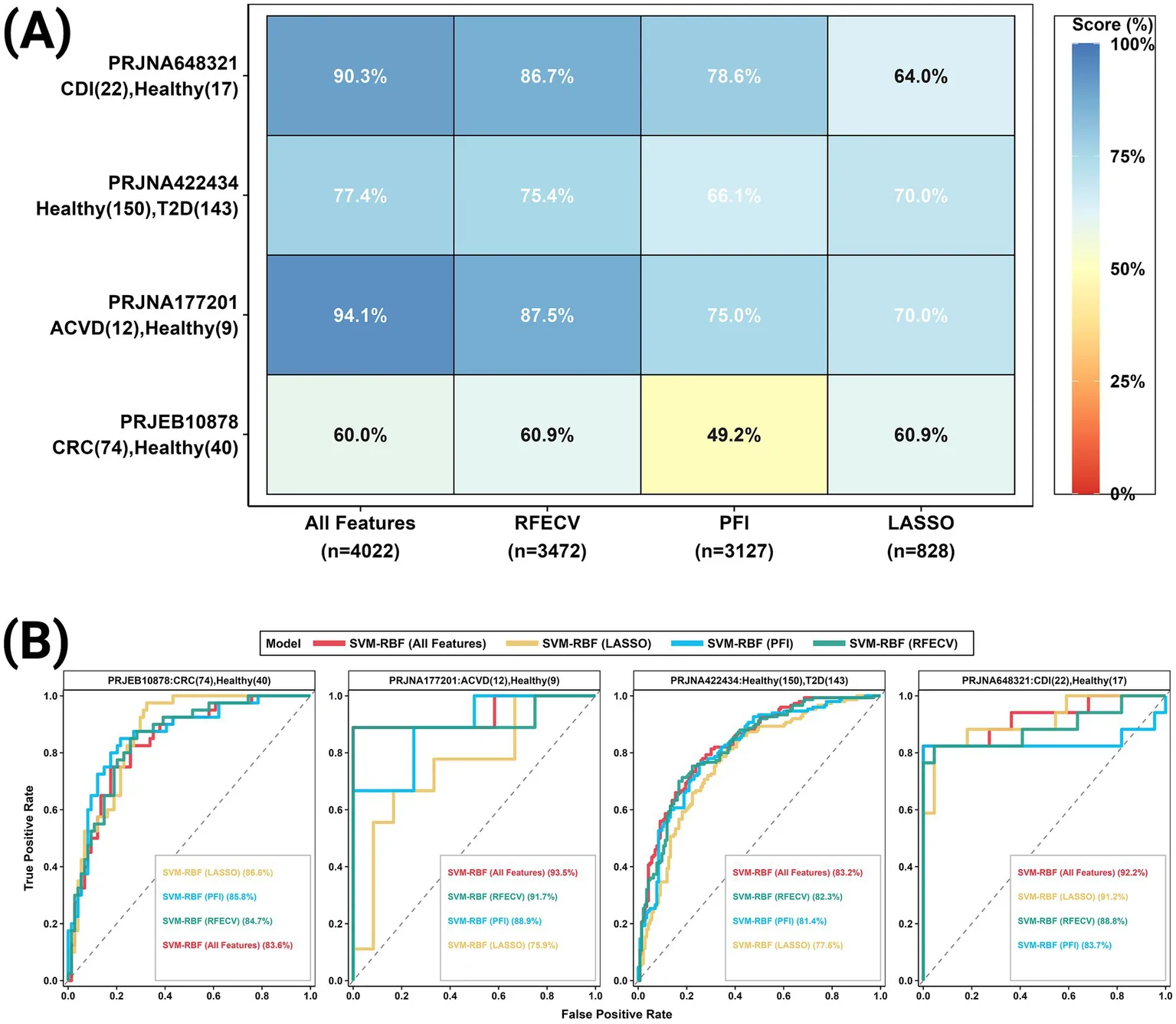
Assessment of SVM-RBF model performance across individual validation studies. Performance metrics (F1 Score and ROC-AUC) are computed separately for each study, with models stratified by feature selection strategy (All Features, LASSO, PFI, RFECV). **(A)** A heatmap shows F1 Scores for each SVM-RBF configuration (rows: All Features, LASSO, PFI, RFECV) across individual validation studies (columns: Study IDs with their phenotype counts). Each cell represents the F1 Score achieved by a specific model on a specific study, with color intensity reflecting performance magnitude (lighter colors indicate lower scores, darker colors indicate higher scores). **(B)** ROC-AUC scores across studies for each model, with the dashed diagonal line representing random classification (AUC = 50%). Note, the F1 and ROC-AUC scores for PRJEB12947 (obesity, *n* = 61) and PRJEB57770 (Parkinson’s disease, *n* = 96) were excluded from both **(A,B)** due to a single class, preventing F1 and ROC-AUC assessment. Performance on these single-class studies is reported using accuracy metrics (Supplementary Figure S9).

To further characterize model performance, we evaluated all SVM-RBF configurations on each disease cohort within the external validation dataset. Our findings indicate that the F1 scores for all models of SVM-RBF ranged from 49 to 94% (Figure 5A), with ROC-AUC scores ranging from 75.9 to 93.5% (Figure 5B) across the validation cohorts. Notably, the SVM-RBF (all features) outperformed the other models, achieving F1 scores between 60 and 90.3% across all disease datasets. Specifically, this model showed best performance on ACVD (F1 score of 94.1% and ROC-AUC score of 93.5%), CDI (F1 score of 90.3% and ROC-AUC score of 92.2%) cohorts, and for T2D (F1 score of 77.4% and ROC-AUC score of 83.2%).

In terms of microbial predictors in the external validation cohort, we examined feature importance profiles across individual disease datasets. The SVM-RBF model identified both shared and cohort-specific microbial taxa contributing to health status predictions (Supplementary Table S12). Our model identified several taxa that were shared across multiple disease datasets, including *Victivallis sp002998355, MGYG000001921, Desulfovibrio sp900319575, Akkermansia muciniphila_B, CAG-269 sp000437215*, and the *Ruminococcus genus*. Among these, *Akkermansia* species is well-established as a health-associated commensal whose depletion is linked to T2D and CDI (Panzetta and Valdivia, 2024; Zhao et al., 2024). Similarly, *Desulfovibrio* species have been consistently reported at elevated abundance across multiple disease states, including PD, OB, and T2D, where they contribute to gut barrier disruption through hydrogen sulphide production (Murros et al., 2021; Nie et al., 2023). The *Ruminococcus genus* encompasses both health-associated fiber-degrading commensals and disease-linked species such as *Ruminococcus gnavus*, which has been implicated in CD (Henke et al., 2019), and metabolic dysregulation (Meadows et al., 2025), consistent with its cross-disease predictive relevance observed here. In addition, we also found cohort-specific taxa (Supplementary Table S12), *Thelissonella pneumosintes* and *Dialister hominis* were prevalent in the CRC dataset, *UCA11452 sp003526375* and *Prevotella sp000431975* were specific to ACVD, *Dialister invisus* and *Acidaminococcus massiliensis* were associated with T2D, and *Bulleidia moorei, Odoribacter splanchnicus*, and *Clostridium species* were specific to CDI. Overall, the SVM-RBF-all features model demonstrated superior performance in the external validation cohorts, suggesting that our ML models more effectively and robustly capture health- and disease-associated microbial patterns when applied to independent datasets.

### 3.6 Evaluation of SVM-RBF features relevance through XAI

Explainable artificial intelligence (XAI) methods can help identify the features driving model predictions. To determine the features contributing most strongly to the predictions of the SVM-RBF model, we calculated SHapley Additive exPlanations (SHAP) values using the Python SHAP library. We considered both the mean absolute SHAP value, which quantifies the overall importance of each feature, and the mean signed SHAP value, which indicates the average direction of its contribution to model predictions (Supplementary Table S15).

**Figure 6:**
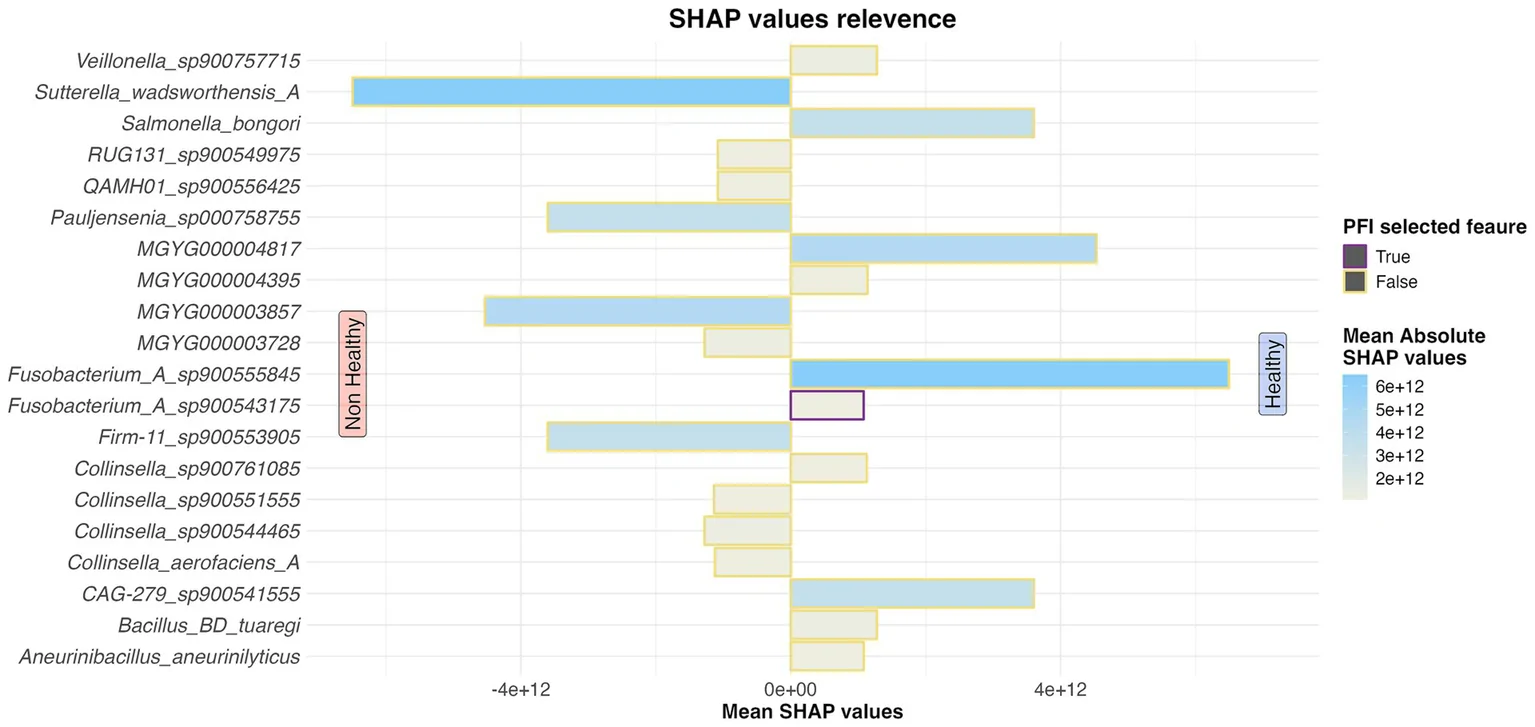
SHAP-based interpretation of the SVM-RBF model. The bar plot displays the 20 features with the highest mean absolute SHAP values. Bar length and direction represent the mean signed SHAP value, with negative values indicating contributions toward the non-healthy class and positive values indicating contributions toward the healthy class. Bar color represents the mean absolute SHAP value, with darker shades indicating greater overall feature importance. Bar borders are colored in green if the feature was also observed in PFI (orchid) or not (yellow).

A total of 2,525 features with mean absolute SHAP values greater than zero were retained. Figure 6 shows the 20 features with the highest mean absolute SHAP values. The direction of each bar indicates the average direction of the feature’s contribution: negative values indicate a contribution toward classification as non-healthy, whereas positive values indicate a contribution toward classification as healthy. The bar color represents the magnitude of the mean absolute SHAP value, with darker shades indicating features with greater overall influence on model predictions.

Accordingly, *Sutterella wadsworthensis* A contributed, on average, toward classification as non-healthy, whereas *Fusobacterium A sp900555845* contributed toward classification as healthy. Among these 20 features, only *Fusobacterium A sp900543175* was also selected by the PFI procedure. Overall, 29 of the 2,525 features retained in the SHAP analysis were also identified through PFI (Supplementary Table S15).

## 4 Discussion

The human gut harbors a dense and taxonomically diverse microbial community whose composition is closely associated to host health and disease (Asnicar et al., 2023; Rosenberg, 2024). In this study, we developed a ML framework to predict gut health status—defined as the presence or absence of a clinically diagnosed disease from species-level stool metagenomic profiles. Classifiers trained on a single cohort tend to learn study-specific technical and population variation rather than microbial signals shared across diseases, which limits their performance when applied to independent cohorts (Li et al., 2023). We therefore pooled 7,452 publicly available stool metagenomes from 32 independent studies covering 12 disease conditions, and trained ML models on this combined dataset to identify dysbiosis signatures that recur across both cohorts and disease types, extending earlier applications of this pooling strategy (Li et al., 2023; Bao et al., 2024; Jin et al., 2024; Lee and Lee, 2024; Defazio et al., 2026).

Our diversity and compositional analyses showed that alpha and beta diversity explain only a small fraction of the disease-associated variation in the gut microbiome (Supplementary Section 1.3). The small effect sizes and inconsistent diversity patterns across diseases indicate that no single diversity metric provides a universal marker of gut health, consistent with previous studies showing that microbiome diversity changes vary across diseases and cohorts (Sun et al., 2024; Williams et al., 2024; Corral López et al., 2026). These findings motivate supervised machine learning on species-level profiles, which can detect multivariate compositional shifts that alpha- and beta-diversity summaries cannot resolve (Kumar et al., 2024; Zhou and Zhao, 2025; Yu et al., 2026).

Pooling large volumes of metagenomic data can introduce study-level batch effects that degrade model performance (Yu et al., 2023). We therefore corrected batch effects using MMUPHin, with BioProject ID as the batch label. Because technical metadata were largely unavailable (Supplementary Section 1.3), *BioProject ID* served as a proxy for methodological differences in library preparation and sequencing, as samples from the same project often share uniform protocols (Barrett et al., 2012). This approach was intended to reduce study-associated technical variation that could compromise cross-cohort generalizability. We acknowledge, however, that BioProject ID may be confounded with disease status, population characteristics, and other biological factors. Consequently, correction based on study identifiers may attenuate genuine biological signals while leaving some residual technical variation. Moreover, disease-associated microbial signatures may themselves be partly attributable to measured or unmeasured confounders (Li et al., 2025). Our analysis therefore focused on reducing inter-study heterogeneity, but it cannot completely separate technical variation from cohort-specific biological variation.

In total, we trained and benchmarked 16 supervised models, combining four algorithms with the full feature set and three feature-selection strategies. In terms of classification performance for predicting health status (healthy vs. non-healthy), the non-linear SVM-RBF model consistently outperformed linear classifiers (SVM-Linear, LogReg-ElasticNet) and RF, both on the held-out test set and in external validation. On the test dataset (*n* = 1,491), the SVM-RBF-PFI model achieved the best performance compared with the full-feature model (F1 = 86.6%, ROC-AUC = 95.5%), with comparably high F1-scores for non-healthy (88.7%) and healthy (86.6%) individuals. Gut metagenomic profiles are high-dimensional, and many features represent rare, low-prevalence taxa detected in only a small proportion of samples. These features may introduce sparse noise rather than reproducible disease-associated information (Papoutsoglou et al., 2023). By retaining only, the species that contributed most strongly to classification, the PFI-based approach may have reduced irrelevant variation and improved the signal-to-noise ratio, thereby enhancing the internal predictive performance of the SVM-RBF-PFI model. Moreover, the SVM-RBF model trained on the full feature set exhibited superior generalization to independent validation cohorts (F1 = 70.6%, ROC-AUC = 84.7%), maintaining robust performance for both healthy (70.6%) and non-healthy (83.0%) individuals, including disease types completely unseen in the training set, such as Clostridioides difficile infection (F1 = 90.3%) and type 2 diabetes (F1 = 77.4%).

We speculate that this trade-off may reflect a broader ecological phenomenon: different bacterial species can perform similar functions in the gut, and a species carrying a disease-associated signal in one cohort may be replaced by a functionally similar species in another (Moya and Ferrer, 2016; Rosenberg, 2024). Such ecological variation has been reported across disease cohorts at scale (Jiang et al., 2025). Restricting the model to species that were most discriminative in the training cohorts may therefore have removed alternative or partially redundant taxa that retained predictive information in independent cohorts and previously unseen diseases. By retaining the complete species set, the full-feature model may have preserved more of these signals. This interpretation is consistent with reports that microbiome biomarkers are often shared across diseases rather than being strictly disease-specific (Duvallet et al., 2017). An ecological perspective has also been used to extend conventional enterotype models. For example, Lai et al. (2023) applied the concept of microbial ecological factors (MEFs) to identify microbial guilds whose collective dynamics were associated with host health (Zhu et al., 2023). Validating models on diseases absent from the training data, as performed here for CDI and T2D, provides a more stringent assessment of cross-disease generalizability than within-cohort cross-validation alone (Steyerberg and Harrell, 2016; Li et al., 2025). he superior performance of the nonlinear RBF kernel relative to the linear classifiers may similarly reflect the complexity of microbial communities, in which taxa interact through processes such as cross-feeding and competition rather than contributing independently (Culp and Goodman, 2023). Consistent with prior work, our results indicate that non-linear SVM-RBF decision boundaries between healthy and non-healthy individuals are more generalizable than linearly constrained boundaries, underscoring its effectiveness in capturing non-linear relationships between microbial community composition and host health status (Kubinski et al., 2022).

Beyond evaluating predictive performance, we identified taxa associated with disease phenotypes and examined the features contributing to model predictions across diverse conditions. Feature-importance rankings, considered alongside MaAsLin2 multivariable association analyses, showed that several influential model features were also statistically associated with host phenotypes. This convergence supports the biological relevance of some of the predictive signals, although it does not exclude residual confounding or other pipeline-related effects. Among the highest-ranked taxa, *Klebsiella pneumoniae* was linked to nine disease states, while *Salinicola tamaricis, Raoultella ornithinolytica*, and *Actinomyces oris* were linked to multiple conditions each—a shared dysbiosis signature rather than disease-specific markers (Supplementary Table S8). Furthermore, taxa such as *Gemella sp002871655, Finegoldia sp900766215, Lactobacillus crispatus, MGYG000003698,* and *Peptoniphilus harei_A* displayed context-dependent associations, being enriched in healthy samples in some comparisons but showing altered abundance or associations in disease in others. These patterns may reflect context-dependent ecological roles in which taxa behave as commensals under some conditions but undergo abundance or functional changes in altered gut environments. They also underscore the dynamic nature of host–microbe and microbe–microbe interactions. Consistent with previous studies, taxa may be associated with both health and multiple disease states rather than being disease-specific (Duvallet et al., 2017; Wilkins et al., 2019; Abbas-Egbariya et al., 2022; Priya et al., 2022; Corral López et al., 2026; da Silva et al., 2026). Such patterns may also arise from strain-level variation, as strains within a species differ in accessory gene content, metabolic capacity, and virulence factors, yielding context-dependent effects on host–microbiome interactions (van der Sommen et al., 2020; Doran et al., 2025). These observations highlight the limitations of species-level interpretation and the need for strain-resolved analyses.

In the case of *Lactobacillus crispatus*, our findings are concordant with previous reports describing its context-dependent role in health and disease (Pan et al., 2020). The model also identified several taxa predominantly associated with health, including *Parvimonas micra, MGYG000003061, Peptoniphilus_A phoceensis, Massilistercora sp902406105, Extibacter hylemonae,* and *Ruthenibacterium lactatiformans*. Notably, although *P. micra* is a normal commensal of the gastrointestinal tract, it is also a well-documented pathobiont enriched in colorectal cancer, periodontitis, and various systemic infections (Xu et al., 2020; Zhao et al., 2022; Higashi et al., 2023). Its association with healthy status in our analysis may reflect its role as a low-abundance commensal organism under healthy conditions. Our training cohort may also not have captured the disease contexts that promote its pathogenic behavior. This is consistent with its role as an opportunistic pathogen that remains harmless in a healthy gut but may contribute to dysbiosis and disease when the gut environment is disturbed (Chow et al., 2011; Kamada et al., 2013; Thomas et al., 2019; Xu et al., 2020; Higashi et al., 2023). Indeed, *P. micra* has been described as an inflammophilic pathobiont whose overgrowth is driven by inflammation and inflammation-damaged tissue (Bergsten et al., 2023). Two main *P. micra* phylotypes (A and B) have recently been described, with phylotype A predominantly associated with colorectal cancer. Variation in *P. micra* abundance was reported at early and late tumor stages but not in adenomas, suggesting that its association with disease may depend on disease stage and local inflammatory conditions (Bergsten et al., 2023).

In external validation datasets comprising entirely independent studies, our model identified both microbial species shared across diseases and taxa specific to particular cohorts or phenotypes (Supplementary File 2). These results suggest that the model did not rely exclusively on patterns associated with the diseases represented in the training data and could recover biologically plausible microbial signatures in previously unseen disease types. *Dialister invisus* and *Acidaminococcus massiliensis* were associated with T2D, while *Bulleidia moorei, Odoribacter splanchnicus,* and *Clostridium species* were specific to CDI. Several of these associations are supported by prior evidence. For example, *Dialister invisus* has been linked to increased intestinal permeability and metabolic dysregulation in diabetic patients (Maffeis et al., 2016; Loaiza et al., 2025), underscoring its relevance to metabolic disease. Similarly, *Odoribacter splanchnicus*, detected specifically in the CDI cohort, has been reported to inhibit *Clostridioides difficile* toxin production through competitive inhibition in clinical and *in vitro* settings, with its abundance negatively correlated with CDI severity (Wang et al., 2026). Collectively, the biological plausibility of these species-level associations across two entirely unseen disease types suggests that the model has learned shared features of gut microbial dysbiosis rather than memorized disease-specific patterns.

Finally, considering its generalization ability we inferred SHAP values on the SVM-RBF model. The returned features partially reprise what we found by combining the SVM-RBF-PFI model with MaAsLin2 analysis. For example, *Sutterella wadsworthensis* A was among the features contributing most strongly toward classification as non-healthy. Other members of this genus were also associated with several disease phenotypes in our analyses. Although *S. wadsworthensis* is commonly present in the gut microbiome and has not itself been characterized as directly pro-inflammatory (Mukhopadhya et al., 2011), recent studies have reported genes encoding proteases capable of targeting IgA1 and IgA2, suggesting a potential mechanism through which it could influence host–microbiome interactions and community composition (Dupraz et al., 2026; Majzoub et al., 2026).

Another taxon contributing toward classification as non-healthy was *Collinsella aerofaciens A*. This observation is consistent with the findings of Chen et al. (2016), who reported enrichment of the genus *Collinsella*, particularly *C. aerofaciens*, in patients with rheumatoid arthritis. Its abundance was correlated with production of the pro-inflammatory cytokine IL-17A, and experiments in an arthritis model supported a role in increasing intestinal permeability. These findings provide biological context for its contribution toward the non-healthy class in our model, although they do not establish the same mechanism across the diverse diseases included here. Interestingly, two *Fusobacterium* species - *Fusobacterium A sp900555845* and *Fusobacterium A sp900543175* - contributed toward classification as healthy. The taxonomy of this genus has recently been extensively revised, revealing substantial genetic heterogeneity among its members; consequently, disease associations reported for some Fusobacterium taxa should not be generalized to all species within the genus (Molteni et al., 2024). Conversely, *P. micra* contributed toward classification as non-healthy, although its mean signed SHAP value was very small (8.9 × 10^−6^). This finding illustrates how model-interpretation methods can reveal subtle predictive contributions, but the small effect magnitude warrants cautious biological interpretation. From a translational perspective, this framework provides a foundation for developing microbiome-based classifiers designed to generalize across multiple disease types rather than being restricted to a single disease. The shared microbial signatures identified here may help prioritize candidates for mechanistic investigation and microbiome-directed interventions and may inform participant stratification in future studies. However, the model classifies samples according to the reported presence or absence of clinically diagnosed disease; it is not a quantitative health index or a substitute for clinical diagnosis. Prospective validation in well-characterized populations, together with standardized demographic, clinical, dietary, medication, technical, functional, and strain-level data, will be necessary before any clinical application can be considered.

### 4.1 Limitations of the studies and future directions

Although the models showed strong predictive performance, the limitations described below should be considered when interpreting these results.

1. The model captures statistical associations between gut microbiome composition and disease-based health classification; it does not establish causality. It should not be used as a clinical diagnostic tool or interpreted as a quantitative index of gut health because it was developed for binary classification rather than individualized health scoring. Its intended purpose is to identify candidate microbial biomarkers and prioritize species for further biological and clinical investigation. Unmeasured host factors—including medication use, diet, age, and BMI—also influence microbiome composition. If these factors are unevenly distributed between the healthy and non-healthy groups, they may confound microbiota–disease associations and generate spurious signals (Vujkovic-Cvijin et al., 2020; Zhu et al., 2026). Our labels follow operational definitions previously used in microbiome health-status frameworks (Gupta et al., 2020; Chang et al., 2024). Because they are based primarily on the reported presence or absence of clinically diagnosed disease, undiagnosed comorbidities, microbiome-altering medication use, and between-cohort biological and technical heterogeneity may introduce label noise, particularly within the healthy group.
2. A direct comparison with frameworks such as MetaML (Pasolli et al., 2016) was not feasible because the models were developed using different taxonomic reference databases and, consequently, different feature spaces and relative-abundance estimates. A valid head-to-head comparison would require reprocessing the same raw sequencing data using harmonized taxonomic profiling, preprocessing, and validation procedures. Without such harmonization, observed performance differences could reflect database and pipeline variation rather than differences between modeling frameworks. To our knowledge, no universally accepted shotgun-metagenomic benchmark dataset currently exists for binary classification of healthy and non-healthy samples. Developing standardized benchmark datasets and evaluation protocols should therefore be a priority for the field.
3. Our model was trained exclusively on bacterial species because bacteria are the best-characterized component of the gut microbiome and the reference database used in this study, UHGG v2.0.2, is designed primarily for prokaryotic profiling (Almeida et al., 2021). The virome and mycobiome were therefore beyond the scope of the present analysis. Future studies incorporating virus- and fungi-specific reference databases could provide a more comprehensive representation of the gut microbial ecosystem.
4. Clinical and demographic variables (e.g., age, sex, BMI) were not used during model training because they were missing for most studies (Supplementary Table S10). Systematic extraction and harmonization of such metadata from published datasets remains an open challenge in the field (Kumar et al., 2024). The same metadata gap meant our batch correction relied on study identity alone, which cannot fully separate technical from biological variation. This limits our ability to fully characterize model behavior, given the influence of host clinical factors on gut microbiota composition (Bajinka et al., 2020) and the potential for unmatched host variables to confound microbiota–disease associations (Vujkovic-Cvijin et al., 2020. Future studies integrating harmonized clinical and demographic metadata with microbial profiles should help disentangle host- and microbiome-driven effects and improve model robustness and interpretability (Wang et al., 2023).
5. Model generalizability was assessed using a limited number of external validation datasets. Although the model retained predictive performance in independent cohorts and disease types not represented during training, validation in larger, more diverse, and prospectively collected clinical populations is required before its translational relevance can be established. Future work should also evaluate a broader range of diseases because dysbiosis signatures may differ among conditions with distinct pathophysiological mechanisms (Kim et al., 2024).
6. The model used species-level abundance profiles and therefore did not capture strain-level variation or functional potential at the gene or pathway level. Strains belonging to the same species can differ in gene content, metabolic capacity, and virulence factors and may consequently exhibit distinct associations with disease despite sharing the same species-level classification. Numerous strain–phenotype and strain–geography associations have been reported across global gut microbiome datasets (Faith et al., 2015; Zhang and Zhao, 2016; Van Rossum et al., 2020; Andreu-Sánchez et al., 2025). Functional changes may also occur without corresponding taxonomic shifts, while pathway profiling can reveal metabolic and ecological relationships that cannot be inferred from taxonomic composition alone (Fuggle et al., 2025; Zielińska et al., 2025). Future work could generate HUMAnN-derived gene-family and pathway profiles as additional model inputs. StrainGE (van Dijk et al., 2022) or StrainScan (Liao et al., 2023) could also be applied to sufficiently prevalent and well-covered biomarker species to determine whether functional or strain-level resolution improves disease-based sample classification and cross-cohort generalization.

One potential extension of our binary classification framework is normative modeling, which quantifies the continuous deviation of an individual’s microbiome from an appropriately defined reference population rather than assigning a binary label (Zhu et al., 2026). Such an approach could potentially improve the characterization of early, intermediate, or subclinical dysbiosis. Two recent frameworks illustrate related strategies. GMWI2 provides a continuous microbiome-based wellness score (Chang et al., 2024), whereas Wiredancer represents the microbiome through multiple continuous ecological factors rather than a single score. The latter framework aims to characterize dysbiotic, protective, and intermediate microbial states and may therefore position samples more precisely along a multidimensional health–disease continuum (Zhu et al., 2026). A complementary direction would be to integrate taxonomic profiles with strain-level and functional information, including pathway and gene-family abundances generated using HUMAnN 3. These microbial data layers could subsequently be combined with other omics data, such as metatranscriptomics and metabolomics, as well as clinical and physiological variables. In disease contexts in which they are relevant, neuroimaging and electrophysiological measurements such as electroencephalography could also be incorporated. Recent large-scale brain–gut cohorts have demonstrated the potential value of combining gut microbiome profiles with neuroimaging, electrophysiological data, peripheral biomarkers, and clinical phenotypes to characterize microbiome–host relationships (Wu et al., 2026). Deep-learning approaches—including Transformers, multilayer perceptrons, and convolutional neural networks—may capture complex microbial patterns as larger and more comprehensively annotated datasets become available (Oh and Zhang, 2020; Su et al., 2022; Bao et al., 2024; Yan B. et al., 2025; Liu et al., 2026). In the present study, however, we deliberately restricted the benchmark to established classical ML algorithms that have been extensively applied to high-dimensional microbiome data (Goodswen et al., 2021; Asnicar et al., 2023). Future studies should compare these methods with appropriately regularized deep-learning architectures using the same cohort-stratified external-validation framework. This would help determine whether more complex architectures provide reproducible improvements in cross-cohort generalization rather than gains confined to the training distribution.

Our study demonstrates that the large-scale integration of publicly available shotgun-metagenomic datasets, combined with standardized processing and explicit management of study-associated variation, can support the development of ML models for gut microbiome-based classification of samples from individuals with and without reported clinically diagnosed disease. The framework is scalable and showed generalization across independent cohorts and disease types absent from the training data. Nevertheless, it is intended primarily as a research framework for cross-disease biomarker discovery and prioritization, not as a clinical diagnostic tool or population-screening instrument. Prospective validation in deeply phenotyped populations, accompanied by harmonized clinical, demographic, technical, functional, and strain-level data, will be required before potential clinical applications can be evaluated.

## Data Availability

Raw sequencing data were retrieved from the NCBI Sequence Read Archive (SRA, https://www.ncbi.nlm.nih.gov/sra). The corresponding BioProject accession numbers and associated metadata are provided in Supplementary Tables S2 and S10. All scripts and features table used for data processing, analysis and models training are available at: https://github.com/bablukr056/ML4GutHealth.

## Footnotes

1
https://github.com/ncbi/sra-tools

2
https://github.com/s-andrews/fastqc

3 Indexed downloaded from: https://genome-idx.s3.amazonaws.com/bt/GRCh38_noalt_as.zip.

4
http://ftp.ebi.ac.uk/pub/databases/metagenomics/mgnify_genomes/human-gut/v2.0.2/kraken2_db_uhgg_v2.0.2/

5
https://github.com/jenniferlu717/KrakenTools/blob/master/combine_kreports.py

6
https://huttenhower.sph.harvard.edu/maaslin/
