# Supplementary material for: A data-driven universal gut microbiome health assessment: a machine learning framework trained on large metagenomic data
Source: Front Microbiol. 2026 Sep 10;17:1925500. doi: 10.3389/fmicb.2026.1925500 (PMC13601785; doi:10.3389/fmicb.2026.1925500)
Supplement: Supplementary file 1 [file Supplementary_file_1.docx]

**Supplementary results**

**1.1 Computational resources utilized for the model development framework**

All the metagenomic data acquisition, curation, preprocessing, and machine learning (ML) model development, and computational workflows (Figure 1B), were executed within standardized and reproducible computational environments (Miniconda v. 25.1.0). Unless otherwise described, all primary analyses were conducted in Python 3.11 using Jupyter Notebook. ML models were implemented with the Scikit‑learn library, while data handling and visualization were carried out using Pandas, NumPy, Matplotlib, SciPy, and Seaborn. Model training was performed on the High‑Performance Computing (HPC) cluster at the ReCaS DataCenter in Bari, Italy, jointly operated by the University of Bari “Aldo Moro” and the National Institute for Nuclear Physics (INFN). Computational tasks were run on nodes equipped with 32 CPUs and 272 GB of RAM, providing adequate resources for large‑scale metagenomic data processing and ML‑based analyses. All figures and statistical analyses presented in the manuscript were generated using R on local workstations configured with 8 CPUs and 16 GB of RAM. These independent R‑based statistical assessments served as an additional validation layer for the findings obtained from the Python‑based workflows.

**1.2 Dataset Overview**

We initially collected a total of 11,492 stool metagenomic samples from 41 published studies through PubMed and Google Scholar. To obtain a high‑quality metagenome, we applied a series of predefined inclusion and exclusion criteria (Methods 2.1). Briefly, we first excluded studies involving antibiotic exposure, participants younger than 18 years, or studies contain samples <10, which removed 2,147 samples from four studies. We subsequently discarded non‑Illumina sequencing datasets, excluding an additional 1,545 samples from five studies. Further filtering eliminated 24 pediatric (age <18 years) individuals based on reported age and 366 low‑quality metagenomes containing fewer than 100,000 classified reads. This sequential curation procedure yielded a final dataset of 7,452 samples from 32 studies. A comprehensive summary of all filtering steps, including excluded studies, the number of removed samples, and the remaining datasets, is provided (*Supplementary Table S1*).

**Supplementary Table S1.** Stepwise filtering and dataset collection overview.

| **Step** | **Filtering criterion** | **Removed datasets (IDs with sample counts)** | **Removed samples** | **Removed studies** | **Remaining samples** | **Remaining studies** |
| --- | --- | --- | --- | --- | --- | --- |
| Initial | Raw dataset | – | – | – | **11,492** | **41** |
| Step 1 | Antibiotic exposed, and <10 samples | PRJEB28237 (842), PRJEB8094 (1224), PRJNA268964 (9) | 2,075 | 3 | 9,417 | 38 |
| Step 2 | Non-Illumina sequencing platforms | PRJEB51776 (592), PRJNA945504 (479), PRJDB3601 (400), PRJNA46321 (56), PRJEB3206 (18) | 1,545 | 5 | 7,872 | 33 |
| Step 3 | Age <18 years | PRJNA821541 (30), PRJNA375935 (2), PRJNA397112 (22) | 54 | 1 | 7,818 | 32 |
| Step 4 | Low read depth (<100,000 classified reads) | PRJNA319574 (200), PRJEB1220 (71), PRJEB4336 (60), SRP057027 (23), PRJEB6337 (6), PRJEB6070 (5), PRJEB19090 (1) | 366 | - | **7,452** | **32** |

*Note: In our study, we finally included 7,452 samples from 32 studies.*

**1.3 Correlation between species and classified reads**

On average, each study achieved a minimum of 80% classified reads (Supplementary Figure 1A) and contributed a mean of 19.04 million classified reads (with a mean of 0.6 million classified sequences per sample, Supplementary Table S3), indicating robust sequencing depth. The mean observed species richness of 2,621 suggests moderate to high microbial diversity. Classified reads are defined as sequences that were taxonomically assigned to known taxa, whereas unclassified reads remained without taxonomic assignment. A strong positive association between species richness and read count was observed using both Pearson (r = 0.73, p < 0.001) and Spearman (r = 0.71, p < 0.001) correlation analyses, supporting the presence of both linear and monotonic relationships (Supplementary Figure 1B). Linear regression analysis indicated that species richness explained 76.33% of the variance in the number of classified reads (R² = 0.7633, p < 0.001), with each additional species corresponding, on average, to an increase of approximately 14,620 classified reads. These findings support that greater sequencing depth improves the detection of both diverse and low-abundance microbial taxa, underscoring its importance for the accurate characterization of complex microbiome communities.

**(A)**

**(B)**

**Supplementary Figure 1.** (A) Horizontal bar plots illustrate the mean number of reads per study, with sample sizes indicated in parentheses (n). Green bars represent taxonomically classified reads that were successfully assigned to known taxa, whereas red bars denote unclassified reads that could not be assigned to any taxonomic category. Study identifiers are ordered by total mean read depth (classified and unclassified) from lowest (bottom) to highest (top). Classification rates exhibit substantial variability across studies, likely reflecting heterogeneity in sample quality, sequencing depth, reference database completeness, and underlying microbial community structure. (B) The scatter plot depicts the association between species richness, and the number of classified sequences reads. Each point corresponds to a single sample, with the x-axis indicating the number of species detected and the y-axis representing the number of classified reads (in millions). The solid green line shows the linear regression fit used to estimate the Pearson correlation coefficient, and the shaded region denotes the 95% confidence interval. The dashed orange line displays a non-linear LOESS smoothing curve corresponding to the Spearman correlation, thereby capturing potential non-linear patterns in the data. Both correlation coefficients are highly significant (p < 0.001, denoted by ***). The R² value indicates the proportion of variance in the number of classified reads that is explained by species richness. In the figure subtitle, (n) denotes the total number of metagenomic samples analysed across all studies.

**1.4 Diversity analysis: Alpha and Beta diversity**

**Alpha diversity**

To characterize the richness and evenness of the human gut microbial community, we computed two alpha-diversity indices, Chao1 (richness) and Shannon (richness and evenness), across 7,452 samples (3,465 healthy and 3,987 non-healthy). Species richness, quantified by the Chao1 index, was significantly higher in the healthy than in the non-healthy group (FDR-adjusted P = 4.16 × 10⁻⁵; Cliff's Δ = 0.055; 95% CI: 0.029–0.081; Supplementary Figure 2A and Supplementary Table S4). Shannon diversity was likewise significantly higher in the healthy group (FDR-adjusted P = 2.07 × 10⁻¹⁴; Cliff's Δ = 0.103; 95% CI: 0.076–0.129; Supplementary Figure 2C and Supplementary Table S4). The two groups were compared using a two-tailed Wilcoxon rank-sum test, and the p-values were corrected for multiple comparisons using the Benjamini–Hochberg false discovery rate (FDR) procedure; effect sizes are reported as Cliff's Δ with 95% confidence intervals. Although both indices differed significantly, the small effect sizes of Cliff's Δ (|Δ| < 0.15) indicate that these differences in richness and evenness are negligible in magnitude.

**Supplementary Figure 2.** Alpha diversity index of gut microbiota comparisons across health status and phenotype. (A) The Chao1 index shows a richness comparison between healthy and non-healthy samples, with healthy individuals showing significantly higher species richness (P < 2.2 × 10⁻¹⁶). (B) Chao1 richness was compared across individual phenotypes, each tested against the Healthy group; richness was significantly reduced in CD, OB, AP, LV, GC, ACVD, and AS (P < 0.01), and increased in PD and CRC (P < 0.05); AMD and UC showed no significant difference. (C) Shannon diversity index compared between healthy and non-healthy samples, showing significantly higher microbial diversity in healthy individuals (P < 2.2 × 10⁻¹⁴). (D) Shannon diversity is compared across individual phenotypes, each tested against the healthy group; diversity is significantly lower in CD, CRC, and LV (P < 0.01), and higher in AMD, PD, ACVD, UC, and GC (P < 0.05); no significant differences are observed in AS, OB, GDM, and AP. Sample sizes are indicated in parentheses below each group label. Statistical significance was assessed using the Wilcoxon rank-sum test with false discovery rate (FDR) correction for multiple phenotype comparisons (B and D): *** P < 0.001, ** P < 0.01, * P < 0.05, ns = not significant. (A) and (C) additionally report Cliff's delta effect size with 95% confidence intervals. Boxes represent the interquartile range (IQR), center lines denote medians, and whiskers extend to 1.5 × IQR. Group-level descriptive statistics (n, mean, SD, median) for both indices are provided by health status and by each of the 12 disease phenotypes (Supplementary Table S4)

Because group sizes were unequal, we performed 100 iterations of random subsampling without replacement from the healthy group, with each subsample matched in size to the disease cohort under comparison. In each iteration, Shannon and Chao1 indices were compared between groups using a two-tailed Wilcoxon rank-sum test, with p-values FDR-corrected across all comparisons. Shannon diversity was significantly lower in AP (P = 1.66 × 10⁻⁷), OB (P < 2 × 10⁻¹⁶), ACVD (P = 4.02 × 10⁻⁹), GDM (P = 0.00664), CD (P < 2 × 10⁻¹⁶), LV (P < 2 × 10⁻¹⁶), GC (P = 0.00109) and AS (P < 2 × 10⁻¹⁶) relative to the healthy group. In contrast, Shannon diversity was higher in PD (P = 1.19 × 10⁻⁷) and CRC (P < 2 × 10⁻¹⁶) than in the healthy group, whereas AMD (P = 0.886) and UC (P = 0.0686) did not differ significantly from the healthy group (Supplementary Figure 2D).

Chao1 species richness showed a partially discordant pattern. CD (P < 2 × 10⁻¹⁶), CRC (P < 2 × 10⁻¹⁶), and LV (P = 1.97 × 10⁻¹⁰) exhibited significantly lower species richness than the healthy group. In contrast, Chao1 richness was significantly higher in AMD (P = 1.91 × 10⁻¹²), PD (P = 3.98 × 10⁻¹²), ACVD (P = 3.16 × 10⁻¹³), UC (P = 1.91 × 10⁻¹²) and GC (P = 8.85 × 10⁻¹³). No significant differences were detected in AP (P = 0.083), OB (P = 0.171), GDM (P = 0.073), or AS (P = 0.468) (Supplementary Figure 2B). The two indices were therefore discordant across several diseases. For example, Shannon diversity was lower in ACVD and GC than in the healthy group, whereas Chao1 richness was higher. Similarly, AMD and UC showed no significant change in Shannon diversity but a significant increase in Chao1 richness. These results are consistent with previous meta-analyses, which report inconsistent associations between alpha diversity and disease status, with very small effect sizes outside diarrheal disease (Walters et al., 2014; Sze and Schloss, 2016; Duvallet et al., 2017).

**Beta diversity**

To assess whether overall gut microbiome composition differs with health status, we quantified beta diversity as the Aitchison distance (the Euclidean distance between centered log-ratio (CLR)-transformed species-level abundances) between the healthy and non-healthy groups. Principal coordinates analysis (PCoA) of these distances showed only limited separation of the two groups (Supplementary Figure 3A). The first two principal coordinates of the PCoA (PC1 and PC2) explained 8.73% and 7.72% of the total variance, respectively (16.45% combined). Nevertheless, PERMANOVA (999 permutations) detected a significant difference in community composition between the two groups (pseudo-F = 41.89; P = 0.001; R² = 0.0056). Health status therefore accounted for only 0.56% of the variation in community composition, indicating a statistically detectable but compositionally marginal effect.

**Supplementary Figure 3.** Principal coordinates analysis (PCoA) of microbiome beta diversity using Aitchison distances (Euclidean distances on CLR-transformed species abundances), showing compositional dissimilarity between healthy and non-healthy samples. **(A)** Each sample is colored by health status — healthy (sky blue) and non-healthy (orange). Compositional differences between both groups were assessed using PERMANOVA (pseudo-F = 41.89; P = 0.001; R² = 0.0056), indicating a statistically significant but very small separation. **(B)** Samples representing six phenotype conditions show significant compositional differences by phenotype (PERMANOVA: pseudo-F = 26.09; P = 0.001; R² = 0.0404), corresponding to 7.2-fold greater explained variance than the binary healthy/non-healthy contrast. In both figures, centroids are shown as filled diamonds, and dashed ellipses denote 95% confidence regions. Sample sizes are indicated in the legend. The first two principal coordinates account for 8.73% and 7.72% of the total variance (16.45% combined).

We then applied PERMANOVA jointly across all 12 disease categories and the healthy group. Phenotype-level classification explained 7.2-fold more variance (pseudo-F = 26.09; P = 0.001; R² = 0.0404) than the binary healthy/non-healthy contrast (R² = 0.0056). This indicates that disease-specific strata capture substantially more compositional variation than a single aggregated non-healthy category (Supplementary Figure 3B).

We therefore performed pairwise PERMANOVA comparisons of each of the 12 disease conditions against the healthy group (Supplementary Table S5). All 12 comparisons were highly significant (p = 0.001 for all), indicating that each disease has a distinct microbiome composition. When diseases were ranked by explained variance (R²), the largest effects were in CD (pseudo-F = 73.44; R² = 0.0186), LV (pseudo-F = 51.89; R² = 0.0141) and CRC (pseudo-F = 42.00; R² = 0.0091), followed by ACVD (R² = 0.0087) and PD (R² = 0.0080). The smallest effects were observed for GDM (pseudo-F = 4.67; R² = 0.0013), AMD (R² = 0.0028) and UC (R² = 0.0032). Overall, these beta-diversity results indicate that compositional differences between health states are statistically significant but small in magnitude (all R² < 0.02), with the most pronounced compositional shifts in Crohn's disease, liver disease and colorectal cancer.

In sum, the alpha- and beta-diversity analyses indicate that most diseases are accompanied by a detectable shift in gut microbiome composition relative to healthy individuals. However, the direction and magnitude of these changes were not consistent across diseases, and the effect sizes (Cliff's Δ and PERMANOVA R²) were generally very small. This is consistent with an earlier large-scale meta-analysis, which likewise reported no uniform reduction in alpha- or beta-diversity across diseases and that the association between disease status and microbial diversity is highly heterogeneous (Duvallet et al., 2017). Disease-specific studies have similarly shown that alpha diversity does not reliably discriminate patients from healthy individuals in most conditions, even after adjusting for potential confounders such as age, sex, BMI, and geographic region. Although beta diversity is sometimes significantly different between groups, the effect sizes are generally too small for either index to serve as a standalone diagnostic marker.

Because alpha- and beta-diversity indices are neither sufficiently discriminative on their own nor consistent across diseases (Williams et al., 2024), we hypothesized that a supervised machine learning approach, which models the joint abundance patterns of thousands of microbial species rather than univariate summary indices, could detect disease-associated microbiome signatures with greater sensitivity and specificity. To test this hypothesis, we developed machine learning models based on SVM-RBF, SVM-Linear, Logistic Regression-ElasticNet (LR-ElasticNet), and Random Forest (RF). All models took species-level abundance profiles derived from stool shotgun metagenomic sequencing as input and were trained to classify each individual as healthy or non-healthy.

**1.5 Batch effect correction in pooled metagenomic cohorts**

Despite the advantages of large-scale pooled analysis, the integration of data from multiple studies can introduce variability across datasets, which may pose challenges to the performance of machine learning models. To account for batch variation arising due to the variation of the studies in the dataset of 7,452 (training/test) metagenomic samples and 642 validation datasets, we applied the batch correction approach to the species count matrix using the R-based MMUPHin package (Method). For clarity, we define each study as a batch. We removed cross-cohort batch effects using the *adjust_batch* function implemented in the MMUPHin R package by using the study ID (i.e., BioProject) as the controlling factor. Batch correction methods are potentially crucial for developing ML models for disease using diverse, multi-cohort data. Before correction of the species count table, PERMANOVA analysis based on Bray–Curtis dissimilarity demonstrates that studies accounted for 12.96% (R² = 0.12962, p = 0.001) and 10.38% (R² = 0.10379, p = 0.001) of microbial community variation in the modelling datasets (n = 7,452) and external validation datasets, respectively. In contrast, health conditions explained only 0.10% (R² = 0.00101, p = 0.001) and 0.51% (R² = 0.00514, p = 0.001) of the variation in the training and validation datasets, respectively. After correction by MMUPHIN, the proportion of variance explained by studies decreased to 6.81% (R² = 0.06807, p = 0.001) in the modelling set and 5.82% (R² = 0.05820, p = 0.001) in the validation set, indicating a significant decrease in batch effects (Supplementary Figure 4A and 4B). Notably, the variance explained by health conditions remained stable or increased slightly (modelling 0.09%, R² = 0.00092; validation 0.61%, R² = 0.00608; p = 0.001), confirming preservation of disease-associated microbial signatures.

**(B)**

**(A)**

**Supplementary Figure 4.** The scatter plot shows the results before and after batch effects correction of the (A) modelling datasets (n=7,542) (B) validation dataset (n=642). In the left panel of both figures, the gamma parameters are explained by the studies. Several batches are marked with different colors. The colored points in the diagram converge to 0 (zero) and increase the similarity of the parameters. The right image of the figures shows the average of the attributes of each batch before and after adjustment. The black points represent the original batch size, and the red points represent the adjusted batch. Each attribute is shown in pairs and connects each point that denotes that attribute in each batch. The contrast of black and red points explains the effect of the adjustment. Red points (adjusted) are oriented closer to the trend line than black points (original), indicating that the adjustment has effectively corrected the batch effect.

In addition to quantifying batch-effect reduction by PERMANOVA, we visualized it by principal coordinates analysis (PCoA) of Bray–Curtis dissimilarities computed from the species-level abundance matrix for the training (n = 7,452) and independent validation (n = 642) datasets, before and after batch correction. The resulting ordinations are shown in Supplementary Figure 5. Before correction, samples from one study in the training dataset formed a discrete cluster at positive values of PCo1, separated from all other studies (Supplementary Figure 5A). After correction, this study-specific clustering was no longer apparent, and samples from different studies were more evenly interspersed in ordination space (Supplementary Figure 5B). This is consistent with the PERMANOVA result, in which study-attributable variance decreased from R² = 0.1296 to R² = 0.0681, a 47.5% relative reduction.

**Supplementary Figure 5.** Graphical representation of PCoA of microbial community structure before and after batch correction. This figure displays PCoA of species-level abundance profiles based on Bray–Curtis dissimilarity. Results from the training dataset (A and B) and the independent validation dataset (C and D) are shown before (A, C) and after batch correction (B, D), respectively. During batch correction, study identity (BioProject) was used as the batch variable. Each point represents an individual sample, while different colours represent different studies (BioProjects). The percentages displayed on the PCo1, and PCo2 axes represent the total variance explained by each principal coordinate.

Before correction, samples formed distinct study-specific clusters, most conspicuously in the training dataset (Supplementary 5A). After correction, this clustering was substantially reduced and samples from different studies were more evenly interspersed in ordination space (Supplementary 5B and 5D). This was consistent with the PERMANOVA-based reduction in study-attributable variance (training dataset R² = 0.1296 to 0.0681; validation datasets R² = 0.1038 to 0.0582; P = 0.001 in both cases).

In the independent validation dataset, no single study formed a pronounced outlier cluster before correction. Samples from different studies partially overlapped, with a small subset separated at negative values of both PCo1 and PCo2 (Supplementary Figure 5C). After correction, samples from different studies were more evenly distributed (Supplementary Figure 5D), although a small residual cluster remained discernible.

This is consistent with the PERMANOVA result for the validation dataset, in which study-attributable variance fell from R² = 0.1038 to R² = 0.0582, a 43.9% relative reduction compared with 47.5% in the training dataset. This residual cluster probably reflects the limits of batch correction in this cohort, given its smaller sample size, the smaller number of contributing studies and the absence of complete technical metadata (see Discussion, Supplementary Table S13). The variance explained by the first two axes of PCoA (PCo1 and PCo2) was similar across all four ordinations (before and after batch correction; training and validation) (training: PCo1 = 11.22% and 12.39%; PCo2 = 5.06% and 4.75%; validation: PCo1 = 10.12% and 10.59%; PCo2 = 7.64% and 7.54%). This indicates that batch correction reduced study-specific clustering while leaving the overall ordination structure largely unchanged. Overall, the PCoA and PERMANOVA results indicate that MMUPHin-based correction substantially reduced study-attributable variance without distorting the overall community structure.

Using study identifiers (i.e. BioProject ID) as the batch label carries an inherent limitation. Because each study was designed around a specific disease phenotype and population, *BioProject ID* is confounded with both disease status and cohort-specific biological variation (Wang and Lê Cao, 2023; Foxx and Rivers, 2025). Statistical studies have shown that when batch and biological group variables (e.g. disease status) are unevenly distributed, batch-correction methods can attenuate true group-level biological differences. As a result, genuine biological signal may be partially removed and downstream inferences may be overconfident (Nygaard et al., 2016).

Batch correction based on study identifiers therefore carries a risk of overcorrection — removal of disease-associated biological signal together with technical noise — which cannot be fully avoided without complete metadata on the sequencing platform, DNA extraction protocol, and library preparation method (Briscoe et al., 2022; Foxx and Rivers, 2025). This is a fundamental limitation of large-scale batch correction in the absence of harmonized technical metadata. Technical variation cannot be removed selectively while fully preserving biologically meaningful between-cohort differences (Austin et al., 2025). A more targeted batch correction strategy, informed by study-level technical metadata, remains an important priority for future large-scale metagenomic studies (Zass et al., 2024).

**1.6 Model Parameters**

**Supplementary Table S6.** Hyperparameter evaluation range for the ML models.

| **Model Type** | **Random search Space** | **All Features**  **(GridSearchCV)** | **PFI**  **(GridSearchCV)** | **RFECV**  **(GridSearchCV)** | **LASSO**  **(GridSearchCV)** |
| --- | --- | --- | --- | --- | --- |
| RF | n_estimators {1000, 1050, 1100, 1150, 1200, 1250, 1300, 1350, 1400, 1450, 1500}, Criterion {Gini, Entropy}, Min Samples Split {2,3,4,5}, Max Features {sqrt, log2}, Bootstrapping {True, False} | max_depth=None, n_estimators=1090, random_state=42 | max_depth=None, n_estimators=1090, random_state=42 | max_depth=15, n_estimators=1140, random_state=42 | max_depth=15, n_estimators=1130, random_state=42 |
| SVM_LIN | C {0.0001, 0.001, 0.01, 0.05, 0.5, 2, 4, 8, 10, 16, 32, 100, 200}, Kernel {linear} | C=0.0001, gamma=scale, kernel=linear | C=0.001, gamma=scale, kernel=linear | C=0.0001, gamma=scale, kernel=linear | C=0.002, gamma=scale, kernel=linear |
| SVM_RBF | C {0.01, 0.05, 0.5, 2, 4, 8, 10, 16, 32, 100, 200}, Gamma {0.0001 … 4, ‘scale’}, Kernel { rbf} | C=20, gamma=scale, kernel=rbf | C=20, gamma=0.0001, kernel=rbf | C=20, gamma=scale, kernel=rbf | C=20, gamma=scale, kernel=rbf |
| LR- ElasticNet | C log-uniform(1e-4,to  1e4), l1_ratio [0,1] | C=0.001274 | C=0.001274 | C=0.001274 | C=0.001274 |

**1.7 Species retention across prevalence filtering thresholds and model performance evaluation (0–25%)**

**(A)**

**Supplementary Figure 6A.** Species retention across prevalence-filtering thresholds (ranging from 0% to 25% in 5% increments) in a pooled microbiome dataset comprising 7,452 samples aggregated from 32 studies. The x-axis denotes the prevalence threshold, defined as the minimum percentage of samples in which a species must be detected to be retained, whereas the y-axis indicates the total number of species remaining after application of each threshold. Each data point is labelled with the corresponding number of retained species and visualized as blue circles. The results demonstrate a progressive reduction in species richness with increasing prevalence thresholds: at 0% prevalence (no filtering), all detected species are retained (n = 4,602); at 5% (n = 4,528); at 10% (n = 4,397); at 15% (n = 4,223); at 20% (n = 4,022); and at 25% (n = 3,799).

**(B)**

**Supplementary Figure 6B.** Comparative performance analysis of four trained machine learning models—SVM-RBF, SVM-Linear, RF, and LR-ElasticNet—evaluated across prevalence thresholds ranging from 0% to 25%. Model performance was assessed on a held-out test dataset comprising 1,491 samples (798 non-healthy and 693 healthy cases) using the F1 score as the primary evaluation metric. Each coloured curve represents the performance profile of a distinct model, with values reported at 5% prevalence increments: SVM-RBF (red), SVM-Linear (dark blue), RF (teal), and LR-ElasticNet (orange). The y-axis denotes the F1 score expressed as a percentage (approximately 75% to 95%), and the x-axis denotes the prevalence threshold as a percentage (0% to 25%). Across all evaluated prevalence thresholds, SVM-RBF consistently exhibited the highest performance, with F1 scores ranging from 85.38% to 86.44%. Furthermore, applying prevalence-based feature filtering improved performance by reducing sparsity and noise, enabling models to capture more consistent microbial signatures associated with disease states, supported by previous studies (Papoutsoglou et al., 2023; Chen et al., 2025). The asterisk (*) denotes the maximum F1 score of 86.44% attained by SVM-RBF at a 20% prevalence threshold, representing the best-performing model–threshold combination. At this threshold, SVM-Linear, LR-ElasticNet, and RF achieved F1 scores of 81.66%, 81.77%, and 79.42%, respectively. Given the superior performance of SVM-RBF at the 20% prevalence threshold, this threshold was selected as the optimal prevalence setting for subsequent analyses and model training.

**1.8 Effect of normalization strategy and CLR pseudo count on model performance**

To evaluate whether the choice of normalization strategy influences model robustness and predictive performance, we compared the performance of our best-performing model (SVM-RBF-PFI) under five preprocessing configurations: raw, untransformed read counts; total-sum scaling (TSS, relative abundance); and centered log-ratio (CLR) transformation at each of three pseudocounts (1.0, 0.5 and 0.1). Furthermore, we applied also the multiplicative replacement method, replacing zeros with a small positive value while proportionally adjusting the non-zero components to preserve the compositional structure and maintain a constant total. To isolate the effect of normalization alone, the same PFI-selected feature set and the same train/test split (n = 5,961 training; n = 1,491 test) were used across all six configurations.

Raw, unnormalized counts performed poorly (F1 = 61.7%, ROC-AUC = 60.5%), consistent with the expectation that untransformed counts carry only relative information and scale with sample-specific library size rather than absolute biological abundance (Gloor et al., 2016; Zhang et al., 2024). TSS normalization, which removes the effect of variable sequencing depth by converting counts to within-sample proportions, improved performance substantially (F1 = 77.6%; ROC-AUC = 86.8%) but leaves the data compositional, and its unit-sum constraint can distort ratios between low-abundance taxa (Gloor et al., 2016; Swift et al., 2023). CLR transformation—the approach used in our primary analysis—outperformed both alternatives (F1 = 86.9%; ROC-AUC = 94.6%; pseudocount = 1.0), consistent with its established use for compositional microbiome data in statistical and machine-learning applications (Gloor et al., 2016). This result, obtained from an independent re-run of our primary model configuration, closely reproduces the values reported in the manuscript (F1 = 86.6%; ROC-AUC = 95.0%), the differences (0.3 and 0.4 percentage points) being consistent with run-to-run variation in model tuning.

**Supplementary Figure 7.** Performance of the SVM-RBF-PFI model on the held-out test set under different normalization strategies. Bar plots show the F1 score (left) and ROC-AUC (right) obtained using five preprocessing approaches: raw counts, total-sum scaling (TSS) relative abundance, and CLR transformation with pseudocounts of 1.0, 0.5 and 0.1. CLR consistently outperformed both raw counts and TSS across both performance metrics, achieving the highest scores at a pseudocount of 1.0 (F1 = 86.9%; ROC-AUC = 94.6%). Performance varied by no more than 1.2 percentage points across the three CLR pseudocounts.

Because CLR requires a logarithm and cannot handle zero counts—which are pervasive in sparse microbiome data—a small pseudocount must be added to all values prior to transformation. We used a pseudocount of 1.0, a common choice in the field, because it preserves sparsity by ensuring zeroes in the input remain zero after transformation. To evaluate the sensitivity of model performance to this choice, we repeated CLR transformation and full model retuning across pseudocounts of 1.0, 0.5, and 0.1 (Supplementary Figure 7). Performance was minimally affected: F1-score varied by only 1.2 percentage points (86.9% to 85.7%) and ROC-AUC by only 0.8 percentage points (94.6% to 93.8%) across this range. Finally, by applying multiplicative replacement we observed a decrease in F1-score (84.47%) and ROC-AUC (94.44%).

In sum these results support two conclusions. First, CLR transformation is the preferred normalization strategy for this framework, clearly outperforming both raw counts and TSS. Second, model performance is stable across a reasonable range of pseudo count values, indicating that our findings are not an artifact of this single parameter choice.

**Supplementary Table S7.** Comprehensive evaluation of classification performance on test datasets across multiple metrics for health status prediction using the all-feature set and feature selection algorithms.

| **Feature-Selection** | **Model** | **F1 Score** | **Balanced Accuracy** | **Accuracy** | **Precision** | **Recall** |
| --- | --- | --- | --- | --- | --- | --- |
| ALL-Features (4,022) | RF | 0.7942 | 0.8074 | 0.8082 | 0.792 | 0.7965 |
|  | SVM-LIN | 0.8166 | 0.8237 | 0.8216 | 0.782 | 0.8543 |
|  | **SVM-RBF** | **0.8644** | **0.8733** | **0.8739** | **0.8644** | **0.8644** |
|  | LogReg-ElasticNet | 0.8177 | 0.8266 | 0.8256 | 0.7954 | 0.8413 |
| RFECV (3,472) | RF-RFECV | 0.7892 | 0.8013 | 0.8015 | 0.7792 | 0.7994 |
|  | SVM-LIN-RFECV | 0.8162 | 0.8232 | 0.8209 | 0.7803 | 0.8557 |
|  | SVM-RBF-RFECV | 0.8569 | 0.8661 | 0.8665 | 0.8539 | 0.86 |
|  | LogReg-ElasticNet-RFECV | 0.8164 | 0.8245 | 0.8229 | 0.7879 | 0.847 |
| PFI (3,127) | RF-PFI | 0.7914 | 0.8047 | 0.8055 | 0.7891 | 0.7937 |
|  | SVM-LIN-PFI | 0.8054 | 0.8155 | 0.8149 | 0.7876 | 0.824 |
|  | **SVM-RBF-PFI** | **0.8657** | **0.8756** | **0.8773** | **0.8806** | **0.8514** |
|  | LogReg-ElasticNet-PFI | 0.8156 | 0.8238 | 0.8223 | 0.7876 | 0.8456 |
| LASSO (828) | RF-LASSO | 0.7977 | 0.809 | 0.8089 | 0.7849 | 0.811 |
|  | SVM-LIN-LASSO | 0.7961 | 0.8049 | 0.8035 | 0.7688 | 0.8254 |
|  | SVM-RBF-LASSO | 0.8553 | 0.8647 | 0.8652 | 0.8534 | 0.8571 |
|  | LogReg-LASSO | 0.8017 | 0.8112 | 0.8102 | 0.7793 | 0.8254 |

**Supplementary Table S11.** Performance of all models on the validation dataset, which includes six independent studies (n = 642 samples).

| **Feature Selection** | **Model** | **F1 Score** | **Balanced Accuracy** | **Accuracy** | **Precision** | **Recall** |
| --- | --- | --- | --- | --- | --- | --- |
| **ALL Features (4022)** | **SVM_RBF** | ***70.64*** | ***78.10*** | ***78.50*** | 65.35 | **76.85** |
|  | LogReg | 60.44 | 68.83 | 66.36 | 50.00 | 76.39 |
|  | SVM_LIN | 59.74 | 67.37 | 61.99 | 46.41 | **83.80** |
|  | RF | 9.61 | 52.31 | 67.76 | **84.62** | 5.09 |
| **RFECV (3472)** | SVM_RBF_RFECV | **68.72** | **76.49** | **77.88** | 65.55 | 72.22 |
|  | SVM_LIN_RFECV | 60.50 | 68.18 | 62.77 | 47.04 | **84.72** |
|  | LogReg_RFECV | 59.85 | 68.24 | 65.73 | 49.40 | 75.93 |
|  | RF_RFECV | 18.11 | 54.51 | 69.00 | **81.48** | 10.19 |
| **PFI (3127)** | ***SVM_RBF_PFI*** | ***60.43*** | ***70.88*** | ***76.95*** | 71.52 | 52.31 |
|  | LogReg_PFI | 52.13 | 61.04 | 59.66 | 43.38 | 65.28 |
|  | SVM_LIN_PFI | 50.47 | 56.51 | 50.47 | 38.03 | 75.00 |
|  | RF_PFI | 18.26 | 54.74 | 69.31 | **88.00** | 10.19 |
| **LASSO (828)** | SVM_RBF_LASSO | **60.91** | **70.46** | **73.21** | 59.82 | 62.04 |
|  | LogReg_LASSO | 57.31 | 66.30 | 65.42 | 49.01 | 68.98 |
|  | SVM_LIN_LASSO | 53.16 | 60.51 | 56.07 | 41.45 | 74.07 |
|  | RF_LASSO | 17.21 | 54.04 | 68.54 | 75.00 | 9.72 |

**Supplementary Figure 8.** ROC-AUC curve for the SVM-RBF classifier retrained on the top 50 highest-ranked species based on feature importance scores from the full-feature model. Our evaluation on the held-out test dataset (n = 1,491; 798 non-healthy, 693 healthy) achieved an ROC-AUC of 88.2%. The ROC-AUC curve (crimson red) demonstrates discriminative ability above random chance (gray dashed diagonal, AUC = 0.50), though this indicates that we found that the SMV-RBF performance declined compared to models trained on all feature sets. The corresponding F1 scores were 77.6% overall, with class-specific scores of 81.0% (non-healthy) and 78.0% (healthy). This decrease in performance suggests that importance-score-based selection alone is not sufficient to capture complex microbial interactions and disease-related signals across the entire feature space, encouraging the subsequent application of ML-based feature selection strategies (LASSO, PFI, RFECV) as described in the main text of the study.

**Supplementary Figure 9.** A heatmap displays Accuracy scores for each feature selection configuration (columns: All Features, RFECV, PFI, LASSO) across individual validation studies (rows: study IDs with their respective phenotype counts). Each cell represents the Accuracy achieved by a specific feature selection method on a specific study, with colour intensity reflecting performance magnitude (lighter colours indicate lower scores, darker colours indicate higher scores). The corresponding F1 Scores for each configuration are reported in the Main Figure 6A.

**Supplementary Table S12.** Top 10 predictive microbial features in the trained SVM-RBF models for each disease cohort in validation datasets.

| **BioProject** | **Feature** | **Model Importance score** |
| --- | --- | --- |
| PRJEB10878  CRC (74) Healthy (40) | *Allisonella_pneumosintes* | 0.008116 |
|  | *Dialister_hominis* | 0.004172 |
|  | *Desulfovibrio_sp900319575* | 0.004058 |
|  | *MGYG000001921* | 0.004058 |
|  | *Victivallis_sp002998355* | 0.004058 |
|  | *Collinsella_sp900552875* | 0.004058 |
|  | *CAG-269_sp000437215* | 0.004058 |
|  | *Ruminococcus_C_sp000433635* | 0.004058 |
|  | *MGYG000000605* | 0.004058 |
|  | *Acidaminococcus_intestini* | 0.002319 |
| PRJNA177201  ACVD (12), Healthy (9) | *MGYG000001921* | 0.039706 |
|  | *Victivallis_sp002998355* | 0.039706 |
|  | *UBA11452_sp003526375* | 0.026471 |
|  | *Prevotella_sp000431975* | 0.026471 |
|  | *CAG-465_sp900554875* | 0.026471 |
|  | *CAG-724_sp003524145* | 0.026471 |
|  | *Ruminococcus_D_bicirculans* | 0.026471 |
|  | *Desulfovibrio_sp900319575* | 0.013235 |
|  | *UMGS268_sp900540175* | 0.013235 |
|  | *CAG-313_sp003539625* | 0.013235 |
| PRJNA422434  Healthy (150), T2D (143) | *Victivallis_sp002998355* | 0.022957 |
|  | *Akkermansia_muciniphila_B* | 0.019513 |
|  | *CAG-170_sp002404795* | 0.019343 |
|  | *PeH17_sp000435055* | 0.019300 |
|  | *Dialister_invisus* | 0.018972 |
|  | *Acidaminococcus_massiliensis* | 0.018548 |
|  | *NK3B98_sp900758315* | 0.018238 |
|  | *Streptococcus_vestibularis* | 0.017264 |
|  | *Gemmiger_sp900556255* | 0.017139 |
|  | *BX12_sp902363595* | 0.017101 |
| PRJNA648321  CDI (21) Healthy (17) | *Akkermansia_muciniphila_B* | 0.036559 |
|  | *Bulleidia_moorei* | 0.036559 |
|  | *Odoribacter_splanchnicus* | 0.036559 |
|  | *Clostridium_P_perfringens* | 0.036559 |
|  | *Clostridium_neonatale* | 0.036559 |
|  | *CAG-269_sp000437215* | 0.036559 |
|  | *CAG-127_sp900319515* | 0.036559 |
|  | *Frisingicoccus_sp900757195* | 0.036559 |
|  | *Ruminococcus_C_sp000980705* | 0.036559 |
|  | *Ruminococcus_C_sp000433635* | 0.036559 |

**Reference**

Austin, G. I., Brown Kav, A., ElNaggar, S., Park, H., Biermann, J., Uhlemann, A.-C., et al. (2025). Processing-bias correction with DEBIAS-M improves cross-study generalization of microbiome-based prediction models. *Nat. Microbiol.* 10, 897–911. doi: 10.1038/s41564-025-01954-4

Briscoe, L., Balliu, B., Sankararaman, S., Halperin, E., and Garud, N. R. (2022). Evaluating supervised and unsupervised background noise correction in human gut microbiome data. *PLoS Comput. Biol.* 18, e1009838. doi: 10.1371/journal.pcbi.1009838

Chen, Y.-C., Su, Y.-Y., Chu, T.-Y., Wu, M.-F., Huang, C.-C., and Lin, C.-C. (2025). PreLect: Prevalence leveraged consistent feature selection decodes microbial signatures across cohorts. *NPJ Biofilms Microbiomes* 11, 3. doi: 10.1038/s41522-024-00598-2

Duvallet, C., Gibbons, S. M., Gurry, T., Irizarry, R. A., and Alm, E. J. (2017). Meta-analysis of gut microbiome studies identifies disease-specific and shared responses. *Nat. Commun.* 8, 1784. doi: 10.1038/s41467-017-01973-8

Foxx, A. J., and Rivers, A. R. (2025). A cautionary tale of batch corrections on confounded microbiome community profiles. *iMetaOmics* 2, e70025. doi: 10.1002/imo2.70025

Gloor, G. B., Wu, J. R., Pawlowsky-Glahn, V., and Egozcue, J. J. (2016). It’s all relative: analyzing microbiome data as compositions. *Ann. Epidemiol.* 26, 322–329. doi: 10.1016/j.annepidem.2016.03.003

Nygaard, V., Rødland, E. A., and Hovig, E. (2016). Methods that remove batch effects while retaining group differences may lead to exaggerated confidence in downstream analyses. *Biostatistics* 17, 29–39. doi: 10.1093/biostatistics/kxv027

Papoutsoglou, G., Tarazona, S., Lopes, M. B., Klammsteiner, T., Ibrahimi, E., Eckenberger, J., et al. (2023). Machine learning approaches in microbiome research: challenges and best practices. *Front. Microbiol.* 14, 1261889. doi: 10.3389/fmicb.2023.1261889

Swift, D., Cresswell, K., Johnson, R., Stilianoudakis, S., and Wei, X. (2023). A review of normalization and differential abundance methods for microbiome counts data. *WIREs Comput. Stat.* 15, e1586. doi: 10.1002/wics.1586

Sze, M. A., and Schloss, P. D. (2016). Looking for a Signal in the Noise: Revisiting Obesity and the Microbiome. *mBio* 7, e01018-16. doi: 10.1128/mBio.01018-16

Walters, W. A., Xu, Z., and Knight, R. (2014). Meta-analyses of human gut microbes associated with obesity and IBD. *FEBS Lett.* 588, 4223–4233. doi: 10.1016/j.febslet.2014.09.039

Wang, Y., and Lê Cao, K.-A. (2023). PLSDA-batch: a multivariate framework to correct for batch effects in microbiome data. *Brief. Bioinform.* 24, bbac622. doi: 10.1093/bib/bbac622

Williams, C. E., Hammer, T. J., and Williams, C. L. (2024). Diversity alone does not reliably indicate the healthiness of an animal microbiome. *ISME J.* 18, wrae133. doi: 10.1093/ismejo/wrae133

Zass, L., Mwapagha, L. M., Louis-Jacques, A. F., Allali, I., Mulindwa, J., Kiran, A., et al. (2024). Advancing microbiome research through standardized data and metadata collection: introducing the Microbiome Research Data Toolkit. *Database* 2024, baae062. doi: 10.1093/database/baae062

Zhang, Y., Schluter, J., Zhang, L., Cao, X., Jenq, R. R., Feng, H., et al. (2024). Review and revamp of compositional data transformation: A new framework combining proportion conversion and contrast transformation. *Comput. Struct. Biotechnol. J.* 23, 4088–4107. doi: 10.1016/j.csbj.2024.11.003
